# The Holistic Life-Crafting Model: a systematic literature review of meaning-making behaviors

**DOI:** 10.3389/fpsyg.2023.1271188

**Published:** 2023-11-23

**Authors:** Llewellyn E. van Zyl, Noah C. M. Custers, Bryan J. Dik, Leoni van der Vaart, Jeff Klibert

**Affiliations:** ^1^Department of Industrial Engineering, University of Eindhoven, Eindhoven, Netherlands; ^2^Optentia Research Unit, North-West University, Vanderbijlpark, South Africa; ^3^Department of Human Resource Management, University of Twente, Enschede, Netherlands; ^4^Department of Social Psychology, Institut für Psychologie, Goethe University, Frankfurt, Germany; ^5^Department of Psychology, Colorado State University, Fort Collins, CO, United States; ^6^Department of Psychology, Norwegian University of Science and Technology, Trondheim, Norway; ^7^Department of Psychology, Georgia Southern University, Statesboro, GA, United States

**Keywords:** life-crafting, meaning-making, job crafting, home-crafting, leisure crafting, systematic literature reviews

## Abstract

Pursuing meaningful life experiences is vital for wellbeing and health. Crafting strategies, such as job crafting and work-life balance crafting, have been developed to create meaning in specific life domains. However, these strategies share common underlying behaviors that transcend specific contexts. Building on this understanding, we propose a comprehensive “holistic life-crafting” approach that integrates overlapping behaviors from various crafting strategies. This study aims to clarify the theoretical conceptualization of life-crafting by identifying common strategies and behaviors underlying different meaning-making crafting approaches. Through a systematic literature search of six databases between January and April 2022, we identified 16,479 published records. Using predefined inclusion–exclusion criteria, 51 records (reflecting five crafting approaches, resulting in 223 different crafting behaviors) remained. Through content analysis, we grouped these behaviors into seven broader crafting strategies, forming the “holistic life-crafting” approach. Findings suggest that life-crafting is a holistic, continuous process of proactive meaning-making by intentionally balancing life demands with available resources and altering life’s cognitive, environmental, interest, relational, skill, and task-related aspects to promote personal growth and wellbeing. The holistic approach encompasses cognitive, environmental, interest, relational, resources-demands, skill, and task crafting strategies. This framework provides a comprehensive understanding of how individuals can actively shape their lives to promote more meaningful life experiences across different domains.

**Systematic review registration:**

PROSPERO, identifier CRD42022333930.

## Introduction

Pursuing meaningful life experiences is a fundamental objective of the human condition and vital for overall wellbeing and flourishing (Jacob and Steger, 2021). Meaning, defined as “the sense that people make of their existence and having an overarching life purpose they pursue” (Steger et al., 2014, p. 27), is an essential antecedent for various positive individual (e.g., happiness), organizational- (e.g., performance), and societal outcomes (e.g., economic prosperity; Jacob and Steger, 2021). Given these benefits, there is increasing interest in understanding the mechanisms and practical approaches to develop meaning (Van Zyl et al., 2010, 2020). According to Jacob and Steger (2021), ‘crafting strategies/approaches’ have become increasingly popular in the literature to help individuals actively create or (re)shape meaningful experiences in different life domains, like work, leisure, or careers. Crafting strategies pertain to a collection of domain-specific behaviors and conscious efforts aimed at changing the physical nature or perception of life/work to better align such with one’s core values, needs, strengths, and goals (Demerouti et al., 2020; Chen et al., 2022).

Largely, the crafting literature developed from industrial/organizational psychology and world of work perspectives, leading to the construction of job crafting (Wrzesniewski and Dutton, 2001). This bottom-up approach helps employees redesign their work experiences by empowering self-directed and value-driven movement toward positive change in meeting complex work demands (Roczniewska et al., 2023). However, researchers and theorists soon started broadening out the concept of crafting to other life domains, including nonwork (De Bloom et al., 2020; Laporte et al., 2021), family (Wan et al., 2021), and leisure (Berg et al., 2010; Kosenkranius et al., 2020) contexts. Currently, there is a strong push to extend the concept of crafting by creating integrative models of meaning-making that cut across different fields of study. In response to these calls, various forms of domain-specific crafting strategies have emerged, ranging from job, home, and career crafting to leisure, story, and work-life balance crafting (Chen et al., 2022). In fact, the literature is saturated with crafting strategies and behaviors, including, but not limited to, cognitive crafting, relational crafting, resource crafting, challenge crafting, demands crafting, task crafting, home crafting, family crafting, leisure crafting, temporal crafting, location crafting, and developmental crafting. These crafting approaches and strategies increase wellbeing and performance in various life domains (Demerouti et al., 2020) but have also been criticized for the fact that their underlying behaviors are stringently context-bound (e.g., crafting in work-related contexts or family life; Chen et al., 2022).

Although meaning arises through domain-specific activities (Messmann and Mulder, 2012; De Jong et al., 2020), human behavior is not entirely context-dependent (Snyder and Tanke, 1976). According to the cognitive consistency theory, people prefer congruence between their thoughts, beliefs, values, attitudes, and behaviors. They are motivated to behave consistently to reduce cognitive dissonance and create coherence in their self-concepts (Kruglanski et al., 2018). Trait activation theory also suggests latent personality traits generate habitual behavioral patterns regardless of context (Tett and Burnett, 2003). Moreover, as creatures of habit, people’s approach to challenges and life tasks is habitual and thus fairly consistent across domains (Snyder and Tanke, 1976; Heimlich and Ardoin, 2008). Deviating from these ingrained, habitual behaviors requires substantial mental effort because they are rooted in deep-seated beliefs, values, personality, skills, enculturation, and socialization (Heimlich and Ardoin, 2008; Wood and Rünger, 2016). Neuroscience also supports the assumption that individuals have a ‘default mode network’ in the brain that reverts thinking and behavior to innate or natural setpoints after situational deviations (De Haan et al., 2023). Therefore, individuals’ underlying psychological makeup and resulting behavioral tendencies remain relatively stable despite their contexts. Hence, crafting strategies may be domain-specific on the surface, but their underlying behaviors are likely to generalize across life domains (Chen et al., 2022). We, therefore, contend that there is an overlap between the different behaviors underpinning different domain-specific crafting strategies. The overlap may lead to a more holistic and encompassing meaning-making or crafting approach: *Life-Crafting*.

Life-crafting is a relatively new concept in the literature referring to a general crafting strategy comprised of universal meaning-making behaviors–but questions remain regarding its conceptualization, operationalization, and measurement (Dekker et al., 2020; Chen et al., 2022). Existing literature presents two approaches to life-crafting. The first approach, described by Schippers and Ziegler (2019), views life-crafting as an intervention framework focused on helping students discover their values/passions, reflect on their present and future competencies/habits-and social lives, consider their ideal future, set concrete goals, and undertake actions to align their values and needs to important areas of life (e.g., social, career, and leisure). Despite its novelty, no empirical evidence of the effectiveness of this approach nor a valid or reliable means to measure underlying concepts was presented. Despite providing a contextual definition for life-crafting, Schippers and Ziegler (2019) neither provide inductive or deductive reasoning for life-crafting as a meaning-making strategy nor link the components to any known meaning-centered theoretical framework. The theoretical basis for the intervention is stated to draw from several different fields (e.g., salutogenesis, positive psychology, goal-setting theory). Still, the connections between these fields and the components of their life-crafting approach are not fully fleshed out (Chen et al., 2022). Chen et al. (2022) echo these concerns by stating that “the conceptual construction of Schippers and Ziegler’s (2019) life-crafting and what it entails is severely lacking” (p. 2). The theoretical grounding and connection to the different components of this approach remain unclear.

Chen et al. (2022) offered an alternative perspective to life-crafting as a collection of meaning-centered behaviors that helps people align their inherent life goals, values, and capabilities to create more meaningful life experiences. Chen et al. (2022) drew from other crafting approaches in the literature to operationalize life-crafting as a foundation for constructing their definition and model and developing/validating a psychometric instrument to measure such. Chen et al. (2022) found evidence and support for a three-factor model of life-crafting. The model defines life-crafting as “the conscious efforts individuals exert to create meaning in their lives through (a) cognitively (re-)framing how they view life, (b) by seeking social support systems to manage life challenges, and (c) to actively seeking challenges to facilitate personal growth” (Chen et al., 2022, p. 23).

While promising, questions remain regarding Chen et al.’s (2022) conceptualization and measurement of life-crafting. First, they identified eight overlapping crafting strategies (positive thinking, personal goal attainment, creating new relationships, optimizing current relationships, utilizing social resources, resources crafting, challenges crafting, and demands crafting), which were later confirmed through qualitative interviews. However, the associated Life-crafting Scale showed that only three factors could be meaningfully extracted: cognitive crafting, seeking social support, and seeking challenges (Chen et al., 2022). Although the authors attempt to provide a psychometric explanation for why these factors did not manifest as intended (e.g., wording effects, shared variance between factors), no theoretical argument was explored for the absence of these factors. It is further unclear why ‘positive thinking’ and ‘goal attainment’ were removed from the overall assessment framework.

Second, the qualitative approach employed to explore the life-crafting strategies was conducted with a sample of Chinese participants and later confirmed within a sample from the Netherlands. However, this approach may have overlooked indigenous meaning-making behaviors specific to the Dutch sample. Because considerable cultural variations exist between Western and Eastern societies in meaning-making, people’s underlying behaviors to create more meaningful life experiences may differ (Wong, 2020; Van Zyl et al., 2023b). This position could explain why only three overarching life-crafting strategies were extracted from the data. Overall, cultural variation in life-crafting strategies represents a significant topic for which additional research is clearly needed to support more inclusive and responsive crafting theories.

Third, the theoretical foundation Chen et al. (2022) used to construct life-crafting is deeply rooted in the conservation of resources theory (Chen et al., 2022). Specifically, life-crafting is primarily focused on preventing the current or future loss of resources. This scope of evaluation is limited and dismisses a plethora of theoretical and empirical evidence suggesting that the function of meaning-making strategies, like life-crafting, is more expansive and integrated (Wong, 2020). For instance, unique features underlying life-crafting serve to support personal growth by increasing engagement in health-coping efforts (Folkman, 1997), expanding access to resilience and well-being resources (Ryff, 2014), bolstering insights on how to find flourishing through suffering (Wong et al., 2021), finding acceptance and reconfiguring ways to “make sense” out of adversity (Park, 2010), and navigating and transcending chaos (Bushkin et al., 2021). Taken as a whole, Chen et al.’s (2022) conceptualization of life-crafting seems restrictive, built mainly around conservation rather than generation or growth, a limitation acknowledged in their work. Moving forward, it will be important to shape life-crafting frameworks by evaluating the intersectionality among different theoretical approaches to meaning-making.

Fourth, the Life-Crafting Scale also posed several psychometric issues. Notably, several items reported significant and large cross-loadings between constructs. These cross-loadings suggest that there may be limited differentiation between life-crafting components (Morin et al., 2020; Van Zyl and Ten Klooster, 2022). Furthermore, the authors do not explain why certain items were dropped from the questionnaire. Chen et al. (2022) only mentioned that three items per construct were essential for parsimony but did not explain or support the process of reducing the item set. The resulting ambiguity risks undermining the conceptualization of life-crafting and its different components, a limitation actively acknowledged by Chen et al. (2022).

Taken together, these limitations provide substantive opportunities to expand the theoretical operationalization of life-crafting. The lack of consensus on the essential elements of life-crafting between Schippers and Ziegler (2019) and Chen et al. (2022), coupled with the conceptual and empirical limitations in both approaches, highlights a need for a more comprehensive, systematic investigation into the theoretical underpinnings of this concept. As such, the present study aims to advance life crafting’s theoretical foundation by identifying common behaviors underpinning different meaning-making or ‘crafting’ approaches/strategies to frame an integrative definition and model of life-crafting.

## Literature review

### Crafting strategies and approaches

Individuals can proactively cultivate meaning through active ‘crafting’, which refers to intentionally changing cognitive, physical, and social features of work, home, or life in general (Wrzesniewski and Dutton, 2001). Wrzesniewski and Dutton (2001) suggested that crafting fosters meaning by helping people (a) assert more control over their lives and avoid alienation, (b) develop a positive self-image, and (c) fulfill basic needs for human connection. This approach, built around the principle of coherence, conveys that meaning-making is a continuous process of creating a closer alignment between the needs/values/strengths of the individual and the requirements of their environment (Wrzesniewski and Dutton, 2001). As such, various forms of domain-specific work (e.g., job [re]crafting) and personal (e.g., home, family, and leisure crafting) crafting approaches and more general crafting strategies (e.g., needs crafting and life-crafting) have emerged in the literature.

*Work-related crafting approaches* foster meaning by helping people consciously reframe the purpose of work, change the nature of work tasks, and create more fulfilling personal relationships at work (Wrzesniewski and Dutton, 2001). *Job crafting* is one of the most popular approaches to creating more meaningful work experiences; it refers to the conscious effort employees initiate to reshape the nature, function, and demands of their work environment to better fit their needs and strengths (Wrzesniewski and Dutton, 2001; Tims and Bakker, 2010). This involves a process by which individuals either change the physical characteristics of their work (i.e., increasing structural job resources, social resources, challenging demands or decreasing hindering job demands; Tims and Bakker, 2010) or reshaping the perceptive boundaries of work-related tasks, relationships, and ways of thinking about work (Wrzesniewski and Dutton, 2001). These proactive strategies help people expand (a) their work’s nature, scope, and boundaries, (b) the meanings they attach to or derive from work, and (c) their professional identities to feel more fulfilled (Wrzesniewski and Dutton, 2001). Job crafting is also directly linked to work engagement, pro-social behaviors, innovation, and higher levels of work-related performance (Bakker et al., 2012; Dubbelt et al., 2019). Because of the noted benefits, job crafting is an important positive psychological intervention strategy to enhance employee wellbeing (Bakker et al., 2012; Van Zyl et al., 2020).

On the other hand, *personal crafting approaches* focus on cultivating meaning in general life domains (Chen et al., 2022). These personal crafting strategies are context-bound, often pertaining to areas outside of work-life, including home crafting, family crafting, leisure crafting, and relational crafting (Chen et al., 2022). Like Tims and Bakker (2010) conceptualization of job crafting, these crafting behaviors draw heavily from the conservation of resources theory. For example, Demerouti et al. (2020) defined *home crafting* as “the changes that employees make to balance their home demands and home resources with their personal abilities and needs to experience meaning and create or restore their person-environment fit” (p. 1013). In deconstructing home crafting, there are several underlying proactive behaviors, including reducing home demands (i.e., behaviors initiated to reduce the mental/emotional/physical load of strenuous activities at home after work), seeking resources (i.e., creating more variety in tasks or asking others for help) and seeking challenges (i.e., being busy at home and looking for activities to stretch one’s capabilities). Essentially, home crafting helps manage negative spillovers from work by generating more positive ‘energy’ at home (Demerouti et al., 2020).

Similarly, Wan et al. (2021) conceptualized *family crafting* as an extension of the job crafting framework. Drawing from the conservation of resources theory, they argued that family crafting refers to behaviors people engage in to create more meaningful family experiences by closing the gap between the family’s needs and capabilities (Wan et al., 2021). Like job crafting and home crafting, family crafting is delineated by three components: reducing hindering family demands and increasing structural family resources and challenging demands. Family crafting highlights one’s ability to alter family-related obligations, relationships, and activities to improve family functioning (De Bloom et al., 2020). Regarding interpersonal outcomes, family crafting strategies buffer against the negative effects of interpersonal conflict on positive family experiences and overall family functioning (Wan et al., 2021).

Theoretically, *leisure crafting* is a unique facet of meaning-making. It draws from the conservation of resources theory but is more concerned with the proactive pursuit of goal setting, human connection, learning, and personal development (Petrou et al., 2017). Petrou and Bakker (2016) argued that leisure crafting helps people change the nature of their hobbies or tasks and reshapes the relational boundaries of their free time. As with other forms of crafting, leisure crafting is (a) proactive and intentional, (b) facilitates personal growth through mastery and challenging demands, and (c) builds companionship and offers new pathways to form relationships (Petrou and Bakker, 2016). Thus, leisure crafting is an important meaning-making mechanism, highly associated with needs for autonomy, competence, and relatedness (Chen et al., 2022).

All these approaches to crafting consider the context (e.g., work or home life) as the primary unit of analysis and define the pathways or behaviors people take to create more meaningful life experiences (Chen et al., 2022). However, a domain-specific approach “violates a more holistic account of human behavior, in which individuals in their work and not work context would be considered [simultaneously]” (Wrzesniewski and Dutton, 2001, p. 196). As such, these domain-specific crafting strategies give credence to developing more *general approaches to crafting*, which consider one’s overall life approach as the unit of analysis (i.e., need-based and life-crafting approaches).

*Needs crafting* refers to the proactive behaviors individuals engage in to consciously manage experiences related to their basic psychological needs, including awareness of personal need satisfaction sources and a propensity to take action based on that awareness (De Bloom et al., 2020). Alternatively, life crafting refers to continuously, holistically shaping life experiences for meaning (Dekker et al., 2020). It is “a process in which people actively reflect on their present and future life, set goals for important areas of life–social, career, and leisure time–and, if required, make concrete plans and undertake actions to change these areas in a way that is more congruent with their values and wishes” (Schippers and Ziegler, 2019, p. 3). Drawing heavily from the conservation of resources theory, an alternative approach to life-crafting argues that it is “conscious efforts individuals exert to create meaning in their lives through (a) cognitively (re-)framing how they view life, (b) by seeking social support systems to manage life challenges, and (c) to actively seeking challenges to facilitate personal growth” (Chen et al., 2022, p. 23). These three approaches provide a set of general meaning-making behaviors which aim to satisfy inherent personal needs, facilitate growth, or help align one’s true-self to life goals.

### Crafting behaviors

Given that most of these approaches draw from the conservation of resources theory, it is not surprising that there is considerable overlap in the associated behaviors people use to craft meaning in different life domains. A brief review of crafting strategies reveals six overlapping meaning-making behaviors: (a) cognitive crafting, (b) relational crafting, (c) task crafting, (d) resources crafting, and (e) demands/challenges crafting.

*Cognitive crafting* involves active efforts to alter how life and work are viewed to be more meaningful (Slemp and Vella-Brodrick, 2013). Specifically, it involves proactive attempts to understand how one’s life or work elements are connected to the success of one’s organization, community, or society (Wrzesniewski and Dutton, 2001). According to Berg et al. (2013), this cognitive framing approach is focused on changing one’s perceptions about one’s boundaries and contribution to society through expanding perceptions, focusing perceptions, and linking perceptions.

*Relational crafting* refers to behaviors that foster meaning by changing how, when, and with whom people interact daily (Berg et al., 2013). These behaviors attempt to establish or maintain high-quality connections or social support systems required to advance their personal or life goals (Berg et al., 2013; Chen et al., 2022). Relational crafting, therefore, aims to expand the quantity or quality of interpersonal connections by building relationships, reframing the nature of current relationships, and adapting the function of relationships (Berg et al., 2013).

*Task crafting* refers to the behaviors designed to physically alter the type, number, scope, and nature of tasks people perform in their daily lives (Wrzesniewski and Dutton, 2001). This process involves the autonomy to add or drop new tasks, alter how tasks are performed, or change how much time/energy is invested in completing various life tasks (Berg et al., 2013). According to Berg et al. (2013), task crafting enhances meaning through three active behaviors: adding additional tasks to work, emphasizing tasks that are energizing or meaningful, and re-engineering or redesigning existing tasks to make them more meaningful (Berg et al., 2013).

Drawing again from the conservation of resources theory, *resource crafting* is a set of proactive behaviors designed to maximize or optimize one’s currently available resources to achieve important work or life goals (Chen et al., 2022). These resources cut across different domains and are valued by people or are needed to achieve important life goals like financial independence, autonomy, or competence (Bakker and Demerouti, 2017). Resource crafters use their abilities, strengths, and preferences to increase or optimize their structural and social resources, enhancing their fit with their environment (Tims et al., 2016; Bakker and Demerouti, 2017). Tims et al. (2012) argued resource crafting has two main components: increasing structural and social resources. Those who resource craft aim to find a balance among available resources, environmental demands, personal abilities, needs, and goals to achieve and flourish (Tims et al., 2016).

Tims and Bakker (2010) noted that people *craft their life demands and challenges* to experience a deeper meaning. *Challenge crafting* refers to the extent to which people engage in activities that challenge their current skills or capabilities to facilitate personal growth, achievement, or accomplishment (LePine et al., 2005). Specifically, challenge crafting helps develop a sense of mastery over a particular life domain through engaging in essential and demanding activities (Chen et al., 2022). Further, where challenge crafting is growth-focused, *demands crafting* seeks to eliminate obstructive or hindering life demands and avoid unnecessary resource loss (Richter et al., 2021; Chen et al., 2022). These hindering life demands are obstacles to achieving individual goals and require substantial effort/energy to overcome (Demerouti and Peeters, 2018). Although these life demands cannot be entirely avoided, demand crafters manage them more effectively by simplifying tasks or making them more efficient (Demerouti and Peeters, 2018). To compensate for these hindering demands, demand crafters proactively seek ways to cope with, eliminate, or avoid the sources of these demands. Prolonged exposure to hindering demands can lead to burnout, stress, and depression (Tims and Bakker, 2010; Demerouti et al., 2020).

### Current study

The marked overlap in behaviors across different crafting strategies, alongside the conceptual limitations of extant life-crafting models (Schippers and Ziegler, 2019; Chen et al., 2022), denote the need to reevaluate and expand our understanding of life-crafting. As such, this systematic literature review aims to broaden the theoretical conceptualization of life-crafting by identifying common elements and behaviors underlying different crafting approaches. Specifically, this study investigates the most common or overlapping crafting behaviors to offer a more holistic, inclusive, and integrative definition and theoretical model for life-crafting. Considering the prevailing literature on crafting, our study addresses a sorely need area of evaluation. Notably, the crafting literature is rather disparate; there is no comprehensive and overarching theoretical groundwork to guide the progression of the field (De Jong et al., 2020). Researchers and theorists constantly develop new crafting dimensions with little consideration for *whether and how such dimensions add value to the literature incrementally*. For example, there are significant challenges in evaluating whether new iterations of crafting are reconfigured expressions of existing crafting concepts or tapping new behaviors and strategies not found within the literature (Chen et al., 2022). In response, our study is one of the first attempts to consolidate this literature base into a meaningful, complex, and cascading crafting model to support future more scientifically framed, nuanced, and integrative research efforts. Once a grounded crafting platform is established, researchers and theorists can tackle other significant limitations (e.g., measurement issues within crafting behaviors; Chen et al., 2022) to solidify further and strengthen this field of study. However, developing theoretical grounding by consolidating and framing the current literature into a meaningful structure or model is of the utmost importance.

## Methodology

### Research approach

An artificial intelligence (AI) assisted systematic literature review was employed to investigate the main objective of this study. AI-assisted systematic literature reviews use active learning to train a predefined machine learning model that forecasts the relevance of records based on specific inclusion/exclusion criteria (Van de Schoot et al., 2021). This approach is particularly relevant when a large number of records are present. The PRISMA 2020 guidelines (Page et al., 2021) steered this systematic literature review (see Appendix G for the PRISMA 2020 checklist). This review was pre-registered on PROSPERO under registration number CRD42022333930.

The decision to implement a systematic review approach was made with consideration for the study’s primary goal and significant scientific, theoretical, and practical limitations within the crafting literature. Notably, the study aimed to evaluate an increasingly complex and expansive literature base and frame results in a digestible manner, especially for busy professionals looking to institute working models of crafting in employment and other settings. Systematic reviews are one of the most transparent, rigorous, and meaningful methods of synthesizing large quantities of data and pinpointing the most relevant aspects of disparate findings across studies (Labarca and Letelier, 2022). They present an overall impression of the quality, limitations, and lingering gaps within a literature base to support more holistic and integrative conclusions and implications, especially for policymakers, program developers, and healthcare providers (Siddaway et al., 2019). Furthermore, they are instrumental in clarifying, paring down, extending, and developing theory (Baumeister, 2003; Booth and Carroll, 2015). These benefits appear aligned well to address some of the more significant limitations within the crafting literature. While crafting is an exciting phenomenon, holding great promise in advancing employee wellbeing (Demerouti et al., 2020), the literature lacks a centered organizational structure to support theory development/extension and bolster the links between scientific evaluation and program development. Essentially, the evaluation of crafting as a scientific concept is proliferating, and there is a great need to locate and define the core components, processes, dimensions, and behavioral expressions underlying life-crafting (Chen et al., 2022). Considering the needs within the literature and benefits of systematic review, we decided to employ a rigorous, modern (AI-assisted), and transparent (pre-registered) evaluation procedure consistent with best practices for systematic reviews (Page et al., 2021).

### Eligibility criteria

At the study’s onset, specific inclusion and exclusion criteria were established to determine record eligibility. Records were eligible for *inclusion* if they: (a) explicitly focused on developing, conceptualizing, or evaluating a novel crafting approach (e.g., job crafting, leisure crafting); (b) explicitly mentioned underlying behaviors of a crafting approach (e.g., task crafting, relational crafting); (c) were published in English or Dutch; (d) appeared in peer-reviewed academic journals or scientific books; and (e) were published between 1997 and 2022.

Records were *excluded* if the: (a) purpose was not explicitly focused on the development, conceptualization, or evaluation of a crafting approach; (b) focus was solely on the antecedents or outcomes of a crafting approach; (c) focus was on behaviors or approaches related to identifying sources of meaning; (d) crafting approach was not positioned as engendering meaningful life/work experiences; (e) focus was on crafting as a creative activity or hobby; (f) the record was not peer-reviewed; (g) record constituted ‘gray literature’; or (h) focus was not on individual behavior.

### Search strategy

This systematic literature search of various bibliometric databases took place in January–April 2022. The following bibliographic databases were used for the search: PsychInfo, LibrarySearch, Web of Science, PubMed, ScienceDirect, and Scopus. Primary and secondary search terms were used to identify relevant literature. Primary terms were: “crafting,” “crafting behavior*,” “crafting strateg*,” “cognitive crafting,” “relational crafting,” “resource* crafting,” “challenge crafting,” “demands crafting,” “life-crafting,” “task crafting,” “job crafting,” “home crafting,” “family crafting,” “leisure crafting,” “temporal crafting,” “location crafting,” “academic leisure crafting,” “crafting toward strengths,” “developmental crafting,” “career crafting,” “collaborative crafting” or “team crafting,” “supervisor rated crafting” or “colleague rated crafting,” and “study crafting.” Secondary terms were: “meaning-making,” “creat* meaning,” “meaning-making strategy,” “meaning crafting,” “strength* use,” “cognitive reframing,” “sense-making,” “mindfulness-to-meaning,” and “expressive emotional coping.” The boolean operators AND/OR were used to combine search terms. These search terms resulted in 31,261 titles from the years 1997 through 2022.

### Selection procedure

The selection process followed a systematic, multi-stage approach with evaluation and expert input. First, two authors executed the search, garnering 31,261 potential records. Descriptive information (e.g., author/s, title, publication title, and publication year) was extracted, and duplicates were removed.

Second, the dataset consisting of 16,479 articles was uploaded in ASReview (2022) (Automatic Systematic Reviews v.0.19), where the titles and abstracts of the articles were independently screened for inclusion using the inclusion and exclusion criteria. ASReview is an AI-assisted tool that employs machine learning to assist in screening large amounts of textual data in systematic reviews (Van de Schoot et al., 2021). The Naïve Bayesian classifier was used with the default TF-IDF feature extraction approach. The initial model was trained by selecting 26 pre-identified relevant records and screening 100 randomly generated irrelevant records. The learning model was run 100 times with the same 100 irrelevant records. Plotted recall curves were generated to visualize the performance of the trained model throughout the entire simulation. Recall curves provided information about the number of publications to be screened and the number of relevant records identified (Van de Schoot et al., 2021). Two authors then re-trained the active learning model’s second iteration based on the first iteration’s labeling decisions to optimize the hyperparameters per topic to receive a more optimal screening model for the convolutional neural network (Van de Schoot et al., 2021). These simulations were then run and screened independently and separately by both authors. Of the 16,479 records, 80% were screened by the first and 51% of the papers by the second author to ensure that all eligible studies after screening were included. ASReview allows identifying 95% of the appropriate papers after screening 33% of the studies (Van de Schoot et al., 2021). In total, 88 relevant records were extracted from the data.

**Figure 1:**
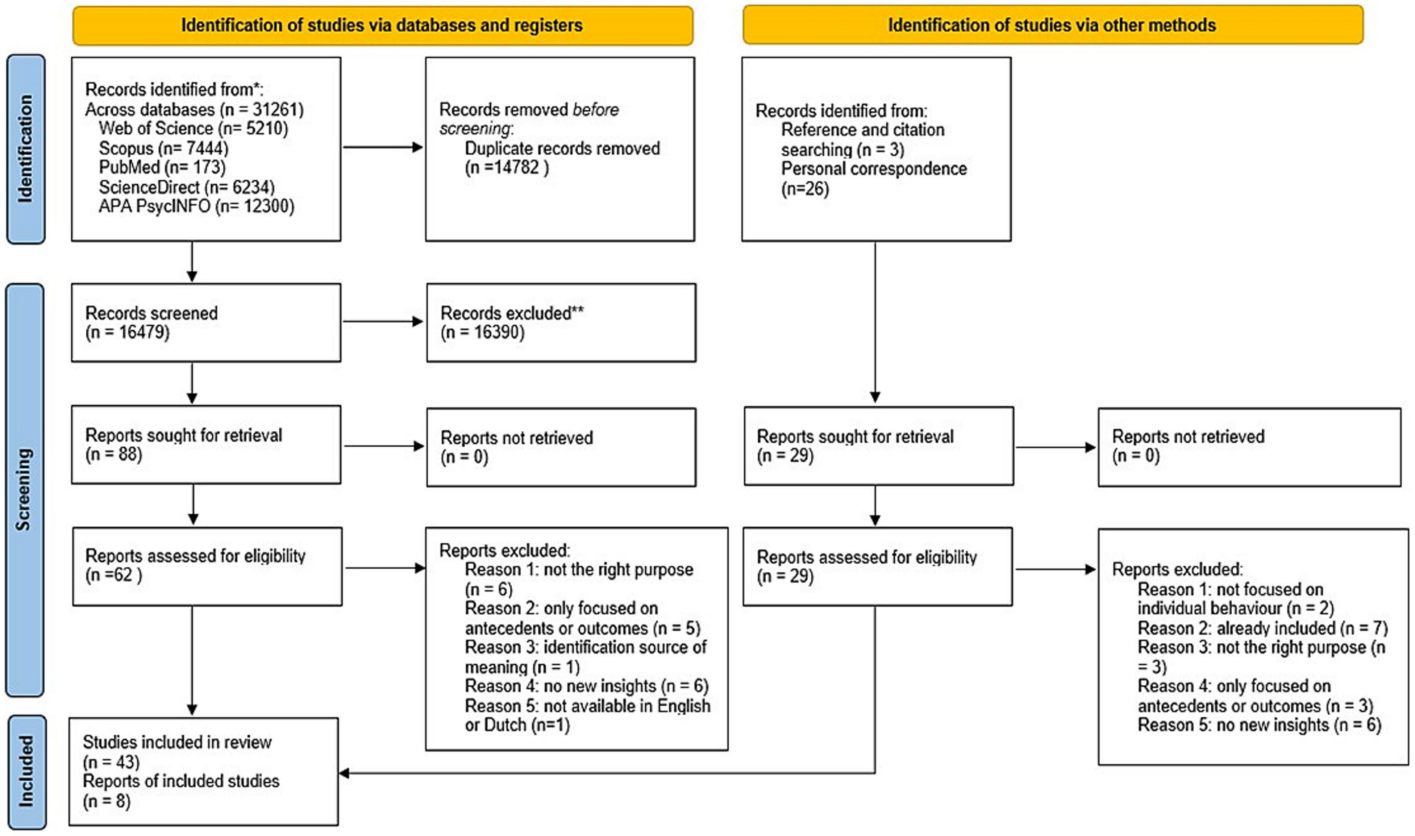
PRISMA flow chart.

Third, these 88 records were further reviewed based on the predefined inclusion/exclusion criteria, excluding 26 further records. A total of 62 articles remained for inclusion. Fourth, full-text records were extracted and evaluated for further consideration. Two authors again screened each record based on the review protocol. After discussing incongruencies, 19 records were excluded for various reasons. Fifth, we screened their reference lists for records that may potentially be relevant. Three additional records were included. After discussions between authors, a final selection of 46 records was made. Finally, the list of final records was distributed to seven experts who provided input on potentially missing records. These academics each had at least 15 years of academic experience, had published at least 10 papers/chapters on crafting, and had published at least 10 papers/chapters related to meaning or meaning-making. Twenty-six additional records were suggested, but only five met the inclusion criteria. Reference and citation searches led to the inclusion of three more articles. In total, 51 records were retained for data extraction. Figure 1 presents the PRISMA flow chart that visually represents the steps taken in the selection process.

### Managing search and reporting bias

Several strategies were used to reduce selection and reporting bias during the review process. First, a clearly defined evaluation taxonomy was constructed before conducting the literature review with specific inclusion/exclusion criteria. The protocol for conducting the search and managing differences was discussed and implemented. Search terms were developed alongside three information management specialists. This taxonomy was strictly followed. Second, all searches were conducted by one author and replicated by another to help ensure no records were missed (Moher et al., 2009; Mohamed Shaffril et al., 2021). Third, two researchers screened and coded all records (titles, abstracts, full texts) independently and in parallel (Buscemi et al., 2006). At each step of the review and selection process, the researchers met to discuss/debate the inclusion/exclusion of the extracted records. The reasons for disagreements were noted, and inter-rater reliability (via Cohen’s Kappa) was calculated for each step of the review process (McHugh, 2012). McHugh (2012) states a kappa coefficient of 0.61 is deemed acceptable. After screening titles and abstracts in our review, Cohen’s kappa coefficient was 0.72, indicating substantial agreement between the raters. After screening the full article records, Cohen’s kappa coefficient was 1, which meant perfect agreement. Fourth, the reference lists of selected records were screened to ensure that all relevant records were included, and backward and forward searches were conducted (Xiao and Watson, 2019). Fifth, the final list of included records was sent to experts to determine if any important records were missed (Foo et al., 2021; Mohamed Shaffril et al., 2021). Sixth, quality assessments (similar to those reported in Van Zyl et al., 2023b) were carried out on each final paper (e.g., the corresponding author’s h-index, the number of paper citations, and the journal’s impact factor). Appendix F (see Supplementary material) shows the mean impact factor of the journals (*M* = 5.60; SD = 3.21) and the medians of the number of citations (71) and h-index of the corresponding author (23).

### Data recording and analysis

Data from the final 51 articles were extracted and captured on a Microsoft Excel spreadsheet for further analysis. Descriptive (e.g., author/s, publication year, and publication type) and content-related information (e.g., research purpose [verbatim], crafting dimensions, and behaviors) about each record was captured and reported. The data was then analyzed through conventional and summative content analysis. This method aimed to find, analyze, and interpret patterns of relevant themes obtained from textual data (Creswell, 2013). The qualitative data analysis followed Miles and Huberman’s (1994) best practice guidelines, unfolding six steps. First, two researchers read all records included in the study to acquire a general overview of types of crafting. Second, they created (both independently and in parallel) the initial codes based on the types of crafting behaviors that were systematically apparent as the data set was processed. Third, each of the two researchers’ codes was grouped (both independently and in parallel) into probable categories based on comparable qualities. Fourth, the researchers compared the themes to the coded extracts to create a thematic map based on the frequency of occurrence. The fifth step (involving all researchers) was a process of ongoing analyzes and constant revision aiming to describe the parts of each topic and guarantee that the overall analyzes tell a coherent story. Disagreements in the coding were discussed until they had been resolved. Finally, themes were compiled based on their frequency and were used to frame a definition of life-crafting. Together, these steps facilitate the trustworthiness of the data analysis process, and all raw process data were retained for scrutiny.

## Results

### Characteristics of included records

The systematic search identified 51 records meeting the inclusion criteria, with most (*n* = 33) published in academic journals during 2018–2022. The records represented five crafting approaches: job crafting (*n* = 32); work-life balance and home crafting (*n* = 6); leisure and off-job crafting (*n* = 5); career and reemployment crafting (*n* = 4); and miscellaneous crafting (*n* = 7). In addition, other descriptive information illustrated some unique characteristics of the retained records. In terms of composition, the retained records included quantitative (*n* = 23), conceptual/theoretical (*n* = 13), qualitative (*n* = 10), and mixed-method (*n* = 5) evaluations. Of the retained empirical/qualitative records (*n* = 38), the majority were conducted in WEIRD (Westernized, Educated, Industrialized, Rich, Democratic) countries (n = 32; 84.2%). Table 1 summarizes the descriptive information.

**Table 1:** Descriptive information of included records.

Authors	Year of publication	Type of paper	Title of paper	Title of journal or book	Type of crafting	Theoretical perspectives	Item type	Country/sample	Purpose*
Berg et al. (2013)	2013	Theoretical	Job crafting and meaningful work	Purpose and Meaning in the Workplace	Job crafting	Cognitive behavior	Book chapter	NA	We [describe] the young and growing literature on job crafting, several ideas for applying job crafting in the workplace to foster meaningfulness, and the various opportunities that exist to build knowledge about how and when job crafting can occur and with what kinds of results.
Berg et al. (2010)	2010	Qualitative	When callings are calling: Crafting work and leisure in pursuit of unanswered occupational callings	Organizational Science	Job crafting Leisure crafting	Cognitive behavior Approach-avoidance Job characteristics–role identities	Journal article	US Educators, NPO and manufacturing employees	Our research suggests that despite the challenges involved in pursuing unanswered callings, individuals can and do exercise agency to do so.
Bindl et al. (2019)	2019	Quantitative	Job crafting revisited: Implications of an extended framework for active changes at work	Journal of Applied Psychology	Job crafting	Cognitive behavior	Journal article	US and UK Miscellaneous University lab participants	We introduce and test an extended framework for job crafting, incorporating individuals’ needs and regulatory focus. Our theoretical model posits that individual needs provide employees with the motivation to engage in distinct job-crafting strategies—task, relationship, skill, and cognitive crafting—and that work-related regulatory focus will be associated with promotion-or prevention-oriented forms of these strategies.
Biron et al. (2023)	2023	Theoretical	Crafting telework: A process model of need satisfaction to foster telework outcomes	Personnel Review	Role-based job crafting	Cognitive behavior	Journal article	NA	The purpose of this study is to offer a model explicating telework as a dynamic process, theorizing that teleworkers continuously adjust – their identities, boundaries and relationships – to meet their own needs for competence, autonomy and relatedness in their work and nonwork roles.
Bruning and Campion (2018)	2018	Mixed-Method	A role-resource approach-avoidance model of job crafting: A multi-method integration and extension of job crafting theory	Business Horizons	Role job crafting	Cognitive-behavior Approach-avoidance Job characteristics–role identities	Journal article	US Miscellaneous	We draw on two studies to develop a role-resource approach-avoidance taxonomy that integrates and extends the dominant role-and resource-based perspectives of job crafting according to characteristics of approach and avoidance.
Caringal-Go et al. (2022)	2022	Qualitative	Work-life balance crafting during COVID-19: Exploring strategies of telecommuting employees in the Philippines	Community, Work, Family	Work-life balance crafting	Cognitive behavior	Journal article	Philippines Telecommuting employees	The purpose of this study is to explore the work-life balance (WLB) crafting strategies of employees with telecommuting work arrangements during the COVID-19 pandemic.
Chen et al. (2022)	2022	Mixed-Method	The life crafting scale: Development and validation of a multi-dimensional meaning-making measure	Frontiers in Psychology	Life-crafting	Cognitive-behavior Approach-avoidance	Journal article	UK and other European countries The Netherlands Miscellaneous	The purpose of this paper was twofold: to conceptualize life-crafting and to develop, validate and evaluate a robust measure of overall life-crafting
De Bloom et al. (2020)	2020	Theoretical	An identity-based integrative needs model of crafting	Journal of Applied Psychology	Crafting within and across life domains	Approach-avoidance Cognitive-behavior	Journal article	NA	With psychological needs satisfaction as the underlying process, we propose an integrative model to account for past conceptualizations of crafting motives and efforts across a person’s various role identities.
De Vos et al. (2019)	2019	Theoretical	From occupational choice to a career crafting	The Routledge companion to career studies	Career crafting	Cognitive-behavior	Book chapter	NA	The objective of this chapter was to provide an overview of how the conceptualization of occupational choice has changed over the past decades, together with a changing perspective on employability and a growing emphasis on the importance of adaptability and career competencies.
Demerouti and Peeters (2018)	2018	Quantitative	Transmission of reduction-oriented crafting among colleagues: A diary study on the moderating role of working conditions	Journal of Occupational and Organizational Psychology	Job crafting	Cognitive-behavior Approach-avoidance	Journal article	The Netherlands Retail and miscellaneous	The goal of this study was to introduce a new form of reduction-oriented job crafting behavior, optimizing demands, and to examine whether and under which conditions the two forms of reduction-oriented job crafting (minimizing and optimizing demands) may be transmitted among colleagues.
Demerouti et al. (2020)	2020	Quantitative	From job crafting to home crafting: A daily diary study among six European countries	Human Relations	Home crafting	Cognitive-behavior Approach-avoidance	Journal article	UK and other European countries Service industry employees	The goal of this study was to uncover whether proactive job crafting behaviors extend to the home domain, and to examine the contextual conditions under which these processes occur.
Dominguez et al. (2019)	2019	Qualitative	Job crafting to persist in surgical training: A qualitative study from the resident’s perspective	Journal of Surgical Research	Job crafting	Cognitive-behavior	Journal article	Colombia Surgical residents	Our study aimed to provide insights into the mechanisms that surgical residents use and value to optimize their demands and resources at the workplace to maintain their engagement and persist in training.
Dreyer and Busch (2021)	2021	Qualitative	At the heart of family businesses: How copreneurs craft work life balance	Journal of Family Business Management	Work-life balance crafting	Cognitive-behavior	Journal article	Germany Copreneurs	The purpose of this paper is to understand how experienced copreneurs of small family business (SFB), as the smallest unit and heart of their family business (FB), may create work-life balance (WLB). Copreneurs evince highly intertwined life-domains and often struggle to respite while managing their high business demands.
Gjerde and Ladegård (2019)	2019	Qualitative	Leader role crafting and the functions of leader role identities	Journal of leadership, organizational studies	Leader role crafting	Job characteristics-role identity	Journal article	Norway Public service, bank/finance, technology sector, and military.	The objective of this study was to explore and provide insights on how experienced leaders address a challenging dilemma between meeting leader role expectations or enacting role in line with role identity, and to theorize the process of leader role crafting which attends to it.
Gravador and Teng-Calleja (2018)	2018	Mixed-Method	Work-life balance crafting behaviors: An empirical study	Personnel Review	Work-life balance crafting	Cognitive-behavior Approach-avoidance	Journal article	Philippines Miscellaneous	The purpose of this paper is to address gaps in the work-life balance (WLB) literature by identifying WLB crafting behaviors employed by individuals, empirically testing which of these behaviors significantly affect WLB, and examining the relationship between the identified WLB crafting behaviors, WLB, and subjective wellbeing (SWB).
Hu et al. (2020)	2020	Theoretical	An exploration of the component validity of job crafting	European Journal of Work and Organizational psychology	Job crafting	Cognitive-behavior Approach-avoidance Job characteristics – role identities	Journal article	NA	This study aimed to clarify these blurred conceptualizations by examining the component and incremental validity of five distinct job crafting measures and their theoretical propositions in predicting work engagement and innovation behavior.
Hulshof et al. (2020)	2020	Quantitative	Reemployment crafting: Proactively shaping one’s job search	Journal of Applied Psychology	Reemployment crafting	Cognitive-behavior Approach-avoidance	Journal article	The Netherlands Unemployed	This article introduces the concept of reemployment crafting: the proactive, self-initiated behaviors undertaken by the unemployed to shape the environmental conditions of their job search in a way that enhances the person– environment (P–E) fit during the job search process.
Jammaers and Williams (2021)	2021	Qualitative	Care for the self, overcompensation and bodily crafting: the work life balance of disabled people	Gender, Work, Organization	Work-life balance crafting and bodily crafting	Cognitive-behavior	Journal article	Belgium Disabled employees	This article argues that studies on work–life balance have neglected the impact of the self-care needs of disabled people in managing their health in and outside the workplace.
Kooij et al. (2022)	2022	Quantitative	Stimulating job crafting behaviors of older workers: The influence of opportunity enhancing human resource practices and psychological empowerment	European Journal of Work and Organizational psychology	Job crafting	Cognitive-behavior Approach-avoidance	Journal article	The Netherlands Retired ‘employees’	We introduced three job crafting behaviors: accommodative, utilization, and developmental job crafting. We hypothesized that opportunity-enhancing HR practices increase psychological empowerment among older workers and therefore their job crafting behavior.
Kooij et al. (2020)	2020	Quantitative	Crafting an interesting job: Stimulating an active role of older workers in enhancing their daily work engagement and job performance	Work, Aging and Retirement	Job crafting	Cognitive-behavior Approach-avoidance	Journal article	The Netherlands Rehabilitation, casino, and online marketing employees	In this diary study, we built on lifespan psychology literature (e.g., Baltes and Baltes, 1990; Carstensen, 1995) and the literature on successful aging at work (e.g., Kooij et al., 2015) to demonstrate that older workers continuously craft their job in such a way that they can do what they find interesting.
Kooij et al. (2015)	2015	Theoretical	Successful aging at work: The role of job crafting	Aging workers and employee-employer relationship	Job crafting	Cognitive-behavior Approach-avoidance	Book chapter	NA	This chapter aims to increase our understanding of older workers as job crafters by drawing upon literature on lifespan development and aging at work to propose specific activities and forms of job crafting relevant for older workers.
Kooij et al. (2017)	2017	Quantitative	Job crafting toward strengths and interests: The effects of a job crafting intervention on person-job fit and the role of age	Journal of Applied Psychology	Job crafting	Cognitive-behavior	Journal article	The Netherlands Health insurance employees	We introduce two novel types of job crafting – crafting toward strengths and crafting toward interests–that aim to improve the fit between one’s job and personal strengths and interests.
Körner et al. (2021)	2021	Quantitative	Study crafting and self-undermining in higher education students: A weekly diary study on the antecedents	International Journal of Environmental Research and Public Health	Study crafting	Cognitive-behavior Approach-avoidance	Journal article	Germany Higher education students	Abstract: The aim of the current study is to validate the adaptation of the job demands–resources theory to the study context. In addition, we introduce the concepts of study crafting and self-undermining to the study demands–resources framework by examining the mediating role of engagement and exhaustion in the relationship between study characteristics and study crafting and self-undermining.
Kosenkranius et al. (2020)	2020	Quantitative	The design and development of a hybrid off-job crafting intervention to enhance needs satisfaction, wellbeing and performance: A study protocol for a randomized controlled trial	BMC Public Health	Off-job crafting	Cognitive-behavior	Journal article	Finland Miscellaneous (knowledge workers)	This article describes the development and design of a hybrid off-job crafting intervention study aimed at enhancing employees’ off-job crafting behaviors, psychological needs satisfaction, wellbeing and performance.
Kroon et al. (2013)	2013	Quantitative	Job crafting en bevlogenheid: Zijn er verschillen tussen teams met een restrictieve dan wel onbegrensde werkcontext?	Gedrag en Organisatie	Job crafting	Cognitive-behavior Approach-avoidance	Journal article	The Netherlands Miscellaneous	In deze studie onderzochten wij twee vormen van job crafting, namelijk uitdagender werk creëren en werkdruk reduceren. (In this study, we examined two forms of job crafting, namely crafting challenging job demands and crafting reduced workload)
Kuijpers et al. (2020)	2020	Quantitative	Align your job with yourself: The relationship between a job crafting intervention and work engagement, and the role of workload	Journal of Occupational Health Psychology	Job crafting	Cognitive-behavior	Journal article	The Netherlands Miscellaneous	This article describes a quasi-experiment that evaluates the relationship between a job crafting intervention and work engagement. More particularly, we focused on three different types of job crafting: crafting toward strengths, crafting toward interests, and crafting toward development.
Laporte et al. (2021)	2021	Mixed-Method	Adolescents as active managers of their own psychological needs: The need crafting in adolescents’ mental health	Journal of Adolescence	Need crafting	Cognitive-behavior	Journal article	Belgium Adolescents	The current research introduces the notion of need crafting, which involves the proactive self-management of need-based experiences. The aim of the present set of two studies is to develop a well-validated and reliable measure for this new concept and to examine its associations with adolescents’ need-based experiences and mental health.
Lazazzara et al. (2020)	2020	Theoretical	The process of reinventing a job: A meta-synthesis of qualitative job crafting research	Journal of Vocational Behavior	Job crafting	Cognitive-behavior Approach-avoidance	Journal article	NA	The goal of this meta–synthesis was to organize the findings from qualitative studies into a process model that highlights when and how people craft their jobs and with what results.
Lee et al. (2021)	2021	Quantitative	Development and validation of the career crafting assessment (CCA)	Journal of Career Assessment	Career crafting	Cognitive-behavior	Journal article	US and Canada Miscellaneous	This research introduces career crafting to describe a set of lifelong career behaviors that people engage in when developing meaningful career paths.
Lichtenthaler and Fischbach (2016)	2016	Quantitative	The conceptualization and measurement of job crafting	Zeitschrift für Arbeits-und Organizations psychologie A&O	Job crafting	Cognitive-behavior Approach-avoidance	Journal article	Germany Police department employees	This research redefined the job demands–resources (JD-R) job crafting model (Tims and Bakker, 2010) to resolve theoretical and empirical inconsistencies regarding the crafting of job demands and developed a German version of the Job Crafting Scale (JCS; Tims et al., 2012).
Lyons (2008)	2008	Qualitative	The crafting of jobs and individual differences	Journal of Business and Psychology	Job crafting	Cognitive-behavior	Journal article	US Sales representatives (consumer products)	This paper examines the concept of spontaneous, unsupervised changes in jobs (job crafting), in general, and the relationship of the qualities and magnitude of the changes to the individual characteristics of: cognitive ability, self-image, perceived control, and readiness to change. This study adds to what is known about individuals at work, in any level of an organization, who knowingly make unsupervised changes in their jobs.
Melo et al. (2021)	2021	Conceptual	Reclaiming cognitive crafting: An integrative model of behavioral and cognitive practices in job crafting	International Journal of Organizational Analysis	Job crafting	Cognitive-behavior	Journal article	NA	This paper aims to present a model of how cognitive and behavioral crafting practices relate, reconciling the two dominant and conflicting job crafting theoretical perspectives.
Nielsen and Abildgaards (2012)	2012	Quantitative	The development and validation of a job crafting measure for use with blue-collar workers	Work, Stress	Job crafting	Cognitive-behavior Approach-avoidance	Journal article	Denmark Mail delivery workers	The contribution of this study is three-fold. First, we were able to confirm the results of previous qualitative research: that is, that blue-collar workers engage in job crafting behaviors. Second, we extended and validated an existing questionnaire on job crafting behaviors, adapting it to a blue-collar context. Third, we identified the types of job crafting behaviors which over time were linked to wellbeing outcomes.
Niessen et al. (2016)	2016	Quantitative	When and why do individuals craft their jobs? The role of individual motivation and work characteristics for job crafting	Human Relations	Job crafting	Cognitive-behavior	Journal article	Germany Miscellaneous	This article focuses on antecedents of job crafting and the development and validation of a job crafting scale.
Petrou and Bakker (2016)	2016	Quantitative	Crafting one’s leisure time in response to high job strain	Human Relations	Employee leisure crafting	Cognitive-behavior Approach-avoidance	Journal article	Multinational Miscellaneous	The present study addresses employee leisure crafting as the proactive pursuit and enactment of leisure activities targeted at goal setting, human connection, learning and personal development.
Petrou et al. (2017)	2017	Quantitative	Weekly job crafting and leisure crafting: Implications for meaning-making and work engagement	Journal of Occupational and Organizational Psychology	Leisure crafting	Cognitive-behavior Approach-avoidance	Journal article	The Netherlands Miscellaneous	The present paper addresses two crafting strategies employees may display in different life domains in order to attain desired outcomes. On the one hand, job crafting is targeted at increasing social and structural job resources and challenging job demands. On the other hand, leisure crafting is the proactive pursuit of leisure activities targeted at goal setting, human connection, learning, and personal development.
Roczniewska et al. (2020)	2020	Qualitative	I believe I can craft! Introducing job crafting self-efficacy scale (JCSES)	PIoS ONE	Job crafting self-efficacy	Cognitive-behavior Approach-avoidance	Journal article	Poland and US Miscellaneous	In this paper we introduced the concept of Job Crafting Self-Efficacy and evaluated the psychometric characteristics of the JCSE Scale.
Rofcanin et al. (2019)	2019	Quantitative	Relational job crafting: Exploring the role of employee motives with a weekly diary study	Human Relations	Relational job crafting	Cognitive-behavior Approach-avoidance Job characteristics – role identities	Journal article	Turkey Employed MBA students	In this weekly diary study, we integrated research on job crafting to explore the associations between expansion and contraction oriented relational job crafting, work engagement and manager-rated employee behaviors (work performance and voice).
Schippers and Ziegler (2019)	2019	Theoretical	Life crafting as a way to find purpose and meaning in life	Frontiers in Psychology	Life-crafting	Cognitive-behavior	Journal article	NA	In this paper, we outlined a life-crafting intervention in which participants complete a series of online writing exercises using expressive writing to shape their ideal future.
Slemp and Vella-Brodrick (2013)	2013	Quantitative	The job crafting questionnaire: A new scale to measure the extent to which employees engage in job crafting	International Journal of Wellbeing	Job crafting	Cognitive-behavior	Journal article	Australia University, banking and finance, and health insurance employees	Empirical research on employee job crafting is scarce, probably because until recently scales with which the construct can be reliably and validly measured were not available. Although a general scale has recently been developed, the cognitive component of job crafting was omitted. The aim of the present study was to address this gap by developing and validating the 15-item Job Crafting Questionnaire (JCQ).
Sturges (2012)	2012	Qualitative	Crafting a balance between work and home	Human Relations	Work-life balance crafting	Cognitive-behavior	Journal article	UK Miscellaneous university graduates	This article reports the findings of a qualitative study that explored the unofficial techniques and activities that individuals use to shape their own work−life balance. It theorizes that this behavior may be usefully conceptualized as physical, relational and cognitive work−life balance crafting.
Tims and Akkermans (2020)	2020	Quantitative	Job and career crafting to fulfill individual career pathways	School to retirement and beyond	Career crafting	Cognitive-behavior Job characteristics –role identities	Journal article	The Netherlands Miscellaneous	The aim of this chapter was to highlight that when individuals know what they want in their jobs and careers, they may engage in proactive behaviors aimed to achieve those personal goals. Individuals may greatly benefit from creating these conditions for themselves, and organizations may also reap the benefits of a highly engaged workforce.
Tims and Bakker (2010)	2010	Theory	Job crafting: Toward a new model of individual job redesign	SA Journal of Industrial Psychology	Job crafting	Cognitive-behavior Approach-avoidance	Journal article	NA	The purpose of the study was to fit job crafting in job design theory.
Tims et al. (2012)	2012	Quantitative	Development and validation of the job crafting scale	Journal of Vocational Behavior	Job crafting	Cognitive-behavior Approach-avoidance	Journal article	The Netherlands Miscellaneous	We developed and validated a scale to measure job crafting behavior in three separate studies conducted in The Netherlands (total *N* = 1,181).
Tsaur et al. (2020)	2020	Mixed-Method	Leisure crafting: Scale development and validation	Leisure sciences	Leisure crafting	Cognitive-behavior Approach-avoidance	Journal article	Taiwan Volunteers and cadres of the Wild Bird Association	The purpose of this study was to develop leisure-crafting dimensions and to design a scale with satisfactory reliability and validity.
Van Wingerden and Niks (2017)	2017	Quantitative	Construction and validation of the perceived opportunity to craft scale	Frontiers in Psychology	Perceived opportunity to craft	Cognitive-behavior	Journal article	The Netherlands Education specialists	We developed and validated a scale to measure employees’ perceived opportunity to craft (POC) in two separate studies conducted in the Netherlands
Weseler and Niessen (2016)	2016	Quantitative	How job crafting relates to task performance	Journal of managerial Psychology	Job crafting	Cognitive-behavior Approach-avoidance	Journal article	Germany Miscellaneous	The purpose of this paper is to investigate the relation between extending and reducing job crafting behavior, cognitive crafting and task performance.
Wessels et al. (2019)	2019	Theoretical	Fostering flexibility in the new world of work: A model of time-spatial job crafting	Frontiers in Psychology	Time-spatial job crafting	Cognitive-behavior	Journal article	NA	We propose a theoretical model of time-spatial job crafting in which we discuss its components, shed light on its antecedents, and explain how time-spatial job crafting is related to positive work outcomes through a time/spatial-demands fit.
Wrzesniewski and Dutton (2001)	2001	Theoretical	Crafting a job: revisioning employees as active crafters of their work authors	Academy of Management Review	Job crafting	Cognitive-behavior	Journal article	NA	We propose that employees craft their jobs by changing cognitive, task, and/or relational boundaries to shape interactions and relationships with others at work.
Yen et al. (2018)	2018	Qualitative	Tour leaders’ job crafting: scale development	Tourism Management	(Tour leaders’) Job crafting	Cognitive-behavior Approach-avoidance	Journal article	Taiwan Tour leaders	The purpose of this study was to develop a scale for measuring tour leaders’ job crafting.
Zhang and Parker (2019)	2019	Theoretical	Reorienting job crafting research: A hierarchical structure of job crafting concepts and integrative review	Journal of Organizational Behavior	Job crafting	Cognitive-behavior Approach-avoidance	Journal article	NA	Two dominant perspectives of job crafting—the original theory from Wrzesniewski and Dutton (2001) and the job demands resources perspective from Tims et al. (2012)—remain separate in research. To synthesize these perspectives, we propose a three-level hierarchical structure of job crafting, and we identify the aggregate/superordinate nature of each major job crafting construct.

*The purpose column contains direct (verbatim) quotes from each record under evaluation. NA, Not applicable.

### Crafting approaches and behaviors

#### Job crafting and perceived opportunity to craft

The majority of records (*n* = 32) concerned job crafting. Appendix A shows the characteristics of the 32 job crafting records. Most records conceptualize job crafting as involving some combination of task, relational, cognitive, physical, skill, promotion-oriented, and prevention-oriented crafting behaviors. These categorized behaviors are used to optimize job resources and demands to create positive meaning or outcomes. Specific approaches like daily crafting, role-based crafting, perceived opportunity to craft, and crafting self-efficacy are also covered.

Looking at the components of job crafting, different perspectives were presented. The first perspective showed that job crafting has three main dimensions: *task crafting, relational crafting,* and *cognitive crafting*. Eight papers referred to these three dimensions (e.g., Berg et al., 2013; Slemp and Vella-Brodrick, 2013; Niessen et al., 2016). *Task crafting* refers to “altering the set of responsibilities prescribed by a formal job description by adding or dropping tasks, altering the nature of tasks, or changing how much time, energy, and attention are allocated to various tasks” (Berg et al., 2013, p. 2). *Relational crafting* refers to “exercising discretion about whom one interacts with at work” (Slemp and Vella-Brodrick, 2013, p. 127). *Cognitive crafting* “comprises re-framing how employees perceive their job and altering their cognitive representation of the job” (Niessen et al., 2016, p. 1289). Berg et al. (2010) used these dimensions but named them *task emphasizing, job expanding,* and *role re-framing*. Biron et al. (2023) used *physical crafting* as a dimension of job crafting instead of task crafting. They described physical crafting as the “active efforts to maintain work-nonwork boundaries and task allocation through managing the quantity, scope, and location of job tasks” (Biron et al., 2023, p. 3). Bindl et al. (2019), p. 607 included *promotion-oriented job crafting* (“approach whereby the employee adds to and extends existing job aspects”) and *prevention-oriented job crafting* (“active changes to one’s job that will prevent negative outcomes from occurring”). Prevention-oriented job crafting does not constitute a withdrawal from work but rather proactive behavior. Bindl et al. (2019), p. 607 furthermore added skill crafting that can occur as *promotion-oriented* (“gaining a wide range of skills through seeking out training opportunities or engaging in stretching assignments/projects”) or *prevention-oriented* (“minimizing failures by focusing on what one does best and optimizing performance in one’s area of expertise”). Lazazzara et al. (2020), Bruning and Campion (2018), Hu et al. (2020), and Melo et al. (2021) used the terms *approach* and *avoidance crafting. Approach crafting* is often defined as attempts “directed toward solving problems, improving the work situation, and accepting and interpreting stressors in a positive way” (Melo et al., 2021, p. 1307). *Avoidance crafting* refers to “efforts to evading, reducing or eliminating parts of one’s work” (Bruning and Campion, 2018, p. 8). Furthermore, Melo et al. (2021), Hu et al. (2020), and Zhang and Parker (2019) described job crafting with the term *behavioral crafting* instead of task and relational crafting. Hu et al. (2020) described *behavioral crafting* as conscious efforts to change the nature of tasks and relationships at work.

Another view on job crafting is finding ways to optimize one’s job resources and manage job demands (Tims et al., 2012). Thirteen records drew from this perspective and classified these behaviors as *increasing structural job resources* (“resources variety, opportunities for development, and autonomy”; Tims et al., 2012, p. 176), *increasing social job resources* (“gaining access to instrumental and emotional support from others and fulfilling their psychological need for relatedness”; Petrou et al., 2017, p. 132), *increasing challenging demands* (“attempts to engage in new activities”; Nielsen and Abildgaard, 2012, p. 376), and *decreasing hindering demands in this dimension* (“efforts to reduce aspects or areas at work which drain energy”; Tims et al., 2012, p. 175). Nielsen and Abildgaard (2012) also included *decreasing social job demands* (“active attempts to avoid emotionally challenging situations”; p. 376) in their job crafting dimensions. Kooij et al. (2015) classified these dimensions into *accommodative crafting* (“crafting activities directed toward regulating losses”; p. 156) and *developmental crafting* (“crafting activities that are directed toward learning new skills or growth”; p. 156). Lichtenthaler and Fischbach (2016) divided the resources and demands dimensions into *promotion-focused job crafting* (increasing resources and challenging demands) and *prevention-focused job crafting* (decreasing hindering demands). Demerouti and Peeters (2018) used the same division of the resources and demands but labeled them *expansion-oriented* (seeking resources and challenges) and *reduction-oriented* (reducing demands). Demerouti and Peeters (2018) and Roczniewska et al. (2020) added *optimizing resources* (“the simplification or optimization of work processes to make them more efficient”; Demerouti and Peeters, 2018, p. 211) to this dimension. Melo et al. (2021) mentioned resources and demands in the *cognitive* and *behavioral crafting practices* within *approach* and *avoidance crafting.*

The results further showed that job crafting might include behaviors where individuals are *crafting toward strengths* (“the self-initiated changes that individuals make in the task boundaries of their work to make better use of their strengths”; Kooij et al., 2017, p. 5) and *crafting toward interests* (sculpting and changing task boundaries at work to access and work within one’s interests; Kooij et al., 2017). Further, Kuijpers et al. (2020) included *crafting toward development* (“the initiatives that employees take to realize their potential by creating developmental opportunities for themselves”; p. 3). Kooij et al. (2020) further explored crafting as a daily activity, finding support for two behaviors: *daily interests* (“the self-initiated changes that individuals make in their work to make it more enjoyable”; p. 165) and the term *daily work pressure* (“the self-initiated changes that individuals make in their work to lower their work pressure”; p. 165) *crafting*.

Finally, Rofcanin et al. (2019) described relational job crafting as a form of job crafting. Relational crafting consists of *expansion-oriented practices* (expanding the type, number, and meaning of interactions employees have with coworkers at work) and *contraction-oriented relational job crafting practices* (contracting the type, number, and meaning of interactions employees have with coworkers at work). Van Wingerden and Niks (2017) described the dimension of *perceived opportunity to craft* (POC), which describes “employees’ perception of their opportunity to craft their job and may determine whether they will proactively craft their job” (p. 1). Wessels et al. (2019) described time-spatial job crafting with the elements of *reflection* (“a deliberate process of thinking about the tasks and private demands and working hours, places, and locations of work available on any particular”; p. 5), *selection* (“the actual choice of working hours, work locations, and workplaces, which is then likely to play a part in reaching the best time/spatial-demands fit”; p. 5), and *adaption* (“performing adaptive behaviors that address changing condition”; Hirschi et al., 2015, p. 1).

#### Work-life balance crafting and home crafting

The characteristics of the six records about work-life balance crafting and home crafting can be found in Appendix B. Five records focused on gaining more insights into work-life balance crafting. In contrast, Demerouti et al. (2020) focused on home crafting using empirical data in a quantitative study. The records about work-life balance crafting used empirical data (qualitative, *n* = 4 and both quantitative and qualitative, *n* = 1). Caringal-Go et al. (2022) focused on employees with telecommuting work arrangements, Dreyer and Busch (2021) focused on co-working couples running their own small family business, and Jammaers and Williams (2021) focused on individuals with a disability. Four records explored strategies, techniques, and/or activities individuals use to shape their work-life balance. Jammaers and Williams (2021) argue that studies on work-life balance have neglected the impact of self-care needs of people with disabilities. The five records yielded their definition of work-life balance crafting, but all were based on existing definitions.

Work-life balance crafting is “proactive, goal-oriented and self-initiated activities to shape boundaries and manage WLB in physical, cognitive, and relational ways” (Dreyer and Busch, 2021, p. 2). Reflecting on the presented dimensions of work-life balance-and home crafting, the results showed that most of the included records shared three components: *physical-, cognitive-,* and *relational crafting. Physical crafting* describes “how work is organized, and it entails joint decisions to change and distribute demands” (Dreyer and Busch, 2021, p. 12). *Cognitive crafting* “involves defining and framing perceptions of what a job means and entails” (Sturges, 2012, p. 1541). Finally, *relational crafting* “involves strategies workers employed to manage both work and non-work relationships” (Caringal-Go et al., 2022, p. 123).

Further, one record included *physical crafting* as a dimension of *work-life balance crafting*. It splits this crafting type into two dimensions: *temporal-and locational (physical) crafting* (Jammaers and Williams, 2021). The paper by Jammaers and Williams (2021) described *physical temporal crafting* as the orienting “around controlling the length of a working day” (p. 122). On the other hand, *Physical locational crafting* is described as the “strategy, locational crafting, employees change the location of their work or home, to cut down the hours needed to get to or physically be present in their standard workplace” (Jammaers and Williams, 2021, p. 122).

Like work-life balance crafting, *home crafting* was found as an additional way individuals craft the nature and function of their home lives. Demerouti et al. (2020) defined home crafting as “changes that employees make to balance their home demands and home resources with their personal abilities and needs, to experience meaning and create or restore their person-environment fit” (p. 1013). These authors distinguished between three types of home crafting behaviors: *seeking home resources* (strategies employed at home to increase the availability of the required resources needed to manage home demands and to achieve goals), *seeking home challenges* (seeking new challenging tasks or taking on more responsibilities once home tasks are completed) and *reducing home demands* (efforts to lessen the emotional, psychological, or physical taxing aspects of home life).

#### Leisure crafting and off-job crafting

The next category of crafting strategies derived from the literature relates to crafting activities in one’s leisure time. The results summarized in Appendix C show that these crafting strategies comprise leisure crafting (*n* = 4) and off-job crafting (*n* = 1). The records about leisure crafting were qualitative (*n* = 1), quantitative (*n* = 2), and mixed-method (*n* = 1) in nature. The off-job crafting paper by Kosenkranius et al. (2020) was a quantitative study, whereas Tsaur et al. (2020) developed a scale for leisure crafting. Three records proposed leisure-crafting dimensions/strategies, and Kosenkranius et al. (2020) developed and designed a framework for off-job crafting. Petrou and Bakker (2016) focused on employee leisure crafting. Berg et al. (2010) described leisure crafting pursuing unanswered callings.

*Leisure crafting* is defined as “the proactive pursuit of leisure activities targeted at goal setting, human connection, learning, and personal development” (Petrou and Bakker, 2016, p. 508). In this respect, two approaches were apparent. First, Berg et al. (2010) focused on *crafting leisure in pursuit of unanswered occupational callings*. This record divided leisure crafting into two dimensions: *vicarious experiencing* (“seeking fulfillment through others’ participation in one’s own unanswered calling”; p. 980) and *hobby participation* (“pursuing leisure and volunteer activities related to an unanswered calling outside of work”; p. 980).

Second, three approaches drew from the conservation of resources theory (Petrou and Bakker, 2016; Petrou et al., 2017; Tsaur et al., 2021) in their conceptualization of leisure crafting. These three records indicated that leisure crafting pertains to efforts associated with increasing resources and managing demands. These authors argued that leisure crafting consists of three dimensions: *increasing social resources, increasing structural resources,* and *increasing challenging demands. Increasing social resources* is described as “gaining access to instrumental and emotional support from others and fulfilling their psychological need for relatedness” (Petrou et al., 2017, p. 132). *Increasing structural resources* is “creating enriched jobs and a motivating job environment” (Petrou et al., 2017, p. 132). Lastly, *increasing challenging demands* is the “increasing feelings of competence and mastery experiences and by creating a challenging environment that promotes growth and learning” (Petrou et al., 2017, pp. 132–133). Tsaur et al. (2021) added *decreasing leisure barriers* as an additional dimension. *Decreasing leisure barriers* refers to “reduce factors hindering leisure participation” (Tsaur et al., 2021, p. 6). Additionally, Petrou and Bakker (2016) argued that leisure crafters actively craft through three activities: *Goal setting* (setting personal goals and creating strategies for actively achieving such through leisure activities), building *human connection* (increasing social contact with others and implementing strategies to develop new human relations during leisure time) and pursuing *learning and personal development opportunities* (seeking growth and development opportunities via leisure activities).

Like leisure crafting, Kosenkranius et al. (2020) introduced *off-job crafting* as a concept that refers to employees’ proactive and self-initiated changes in their non-working lives to satisfy their psychological needs. From this perspective, off-job crafting comprises of six proactive behaviors: *crafting for detachmen*t (“mentally disengaging from work-related matters”), *crafting for relaxation* (“proactively striving for feeling physically well and for reducing effortful activities”), *crafting for autonomy* (“striving for a feeling of being in control over one’s actions, life, and choices”), *crafting for mastery* (“seeking learning opportunities and optimal challenges to experience feelings of achievement and competence”), *crafting for meaning* (“engaging in activities that individuals perceive as opportunities to gain something valuable in life”), and *crafting for affiliation* (“the desire to experience relatedness and belongingness with other people”; Kosenkranius et al., 2020, p. 2).

#### Career crafting and reemployment crafting

Career crafting and reemployment crafting emerged as distinctive crafting strategies individuals employ to facilitate career progression or to gain meaningful employment. Appendix D summarizes the different perspectives relating to career- (*n* = 3) and reemployment crafting (*n* = 1). De Vos et al. (2019) provided an overview of career crafting, while Lee et al. (2021) introduced a career crafting assessment, and Hulshof et al. (2020) introduced the concept of reemployment crafting.

*Career crafting* is defined as “a set of proactive and congruence-seeking behaviors that (a) broadens career-relevant resources in response to the evolving nature of jobs and (b) explores career options more congruent to one’s changing needs, values, and interests” (Lee et al., 2021, p. 718). Lee et al. (2021) divided *career crafting* into three dimensions: *career-level task, career-level relationship,* and *career-level cognition crafting*. *Career-level task crafting* is “the practice of changing the type, scope, and number of job tasks to suit an individual’s strengths and values better” (Lee et al., 2021, p. 718–719), which includes *expanding task boundaries* (“take on extra tasks to experience new career-related responsibilities in their organization”; Lee et al., 2021, p. 731)*. Career-level relationship crafting* is defined as changes in “the amount and quality of interactions with other people encountered on the job” (Lee et al., 2021, p. 719) and includes *changing relational boundaries* (“the vital role of proactive relational crafting in producing positive career outcomes”; Lee et al., 2021, p. 731) and *utilizing relational resources* (“the vital role of proactive relational crafting in producing positive career outcomes”; Lee et al., 2021, p. 731). *Career-level cognition crafting* “involves altering the individual’s perception of their work, such as interpreting their job as a part of fulfilling their life story instead of viewing work as a means of living” (Lee et al., 2021, p. 719). This form of crafting includes *reflecting positive career meaning* (“indicating that career crafters view their careers as a significant part of their life”; Lee et al., 2021, p. 731).

Tims and Akkermans (2020, p. 14) explained that *proactive career behaviors* “should allow individuals to achieve life and career success.” Tims and Akkermans (2020) split proactive career behaviors into *proactive career reflection* and *construction*. *Proactive career reflection* represents “individuals who proactively reflect on their career motivations and skills (e.g., on motivations and qualities; p. 22).” *Proactive career construction* reflects “individuals who proactively try to advance their careers by networking, may be more likely to achieve careers they find fulfilling [e.g., networking and setting goals]”; (Tims and Akkermans, 2020, p. 22).

In their approach to career crafting, De Vos et al. (2019) described career crafting as an “Individual’s proactive behaviors aimed at optimizing career outcomes through improving person-career fit” (p. 129). Individuals should actively craft their careers over time by (a) *reflecting on and being mindful of their career aspirations and motivation* and (b) *making choices that can impact both short-term and long-term success.*

Finally, *reemployment crafting* was introduced as a set of behaviors and strategies for the unemployed and drew from the conservation of resources theory. *Reemployment crafting* is described as “the proactive, self-initiated behaviors undertaken by the unemployed to shape the environmental conditions of their job search in a way that enhances person-environment (P-E) fit during the job search process” (Hulshof et al., 2020, p. 58). *Reemployment crafting* consists of three dimensions: *seeking resources* (the individual’s personality, social support, financial resources, and ability to structure one’s time during unemployment)*, reducing hindering demands* (minimizing those aspects of the job search that exceed one’s capabilities), and *seeking challenging demands* (creating more positively interpreted demands to feel motivated to continue one’s job search).

#### Miscellaneous crafting types

Crafting strategies and behaviors that could not be classified into the aforementioned categories were classified as miscellaneous crafting types. These crafting strategies represent new or emerging research fields, the characteristics of which are summarized in Appendix E. This category includes *life-crafting* (*n* = 2), *leader role crafting* (*n* = 1), *study crafting* (*n* = 1), *needs crafting* (*n* = 1), *bodily crafting* (*n* = 1), and *crafting within and across life domains* (*n* = 1). The records relating to *leader role crafting, bodily crafting*, and *study crafting* were qualitative, whereas those describing *needs crafting* and *life-crafting* employed mixed-method approaches. The final *life-crafting* record and the record about *crafting within and across life domains* were theoretical. Three records introduced new concepts, and two records provided more insights into an already-known concept. Two records developed and validated a measure for a concept, and one validated the adaption of the job demands-resources theory. De Bloom et al. (2020) proposed a model to account for past conceptualizations of crafting motives.

*Bodily crafting* is described as “the unofficial techniques and activities disabled employees use to work on their bodies and keep fit for both work and non-work purposes to better articulate life and work–to better grasp the embodied experience of a neglected group of workers” (Jammaers and Williams, 2021, p. 120). Jammaers and Williams (2021) mentioned two dimensions of *bodily crafting,* namely *cognitive crafting* (“employees redefining what WLB means to them”; p. 122) and *relational crafting* (“building good relationships with key people in one’s environment, both inside and outside the workplace, to establish a better balance”; p. 122).

*Study crafting* is described as “the proactive changes that students make in their study demands and study resources, and therefore the active influence of the student on his or her study environment” (Körner et al., 2021, p. 14). Study crafting consists of *increasing structural and social resources* and *limiting study demands. Increasing structural resources* are behaviors that influence the study’s design. *Increasing social resources* is the social aspect of one’s study and consists of *social support from lecturers* and *social support from fellow students. Limiting study demands* are concerned with the psychological, physical, social, or organizational study aspects that require effort and are associated with mental or physiological costs (Körner et al., 2021).

*“Life-crafting* is about (1) finding out what you stand for (i.e., values and passions), (2) finding out how to make it happen (i.e., goal-attainment plans), and (3) telling someone about your plans (i.e., public commitment; Schippers and Ziegler, 2019, p. 12).” Seven steps were designed to craft lives. These steps are (1) *discovering values and passion,* (2) *reflecting on current and desired competencies and habits,* (3) *reflecting on present and future social life,* (4) *reflecting on a possible future career,* (5) *writing about the ideal future,* (6) *writing down specific goal attainment and “if-then” plans,* and (7) *making public commitments to the goals set.*

Chen et al. (2022) described *life-crafting* as “conscious efforts individuals exert to create meaning in their lives through (a) cognitively (re-)framing how they view life, (b) by seeking social support systems to manage life challenges, and (c) to actively seeking challenges to facilitate personal growth” (p. 1). *Cognitive crafting* is the “individual’s ability to proactively reshape or cognitively re-frame the physical, cognitive or social features of work or life in order for it to be perceived as more meaningful” (p. 1). *Seeking social support* is defined as “the extent to which individuals seek out social support systems and networks to achieve personal/professional goals and aid in managing adversity” (p. 1). *Seeking challenges* is “the active efforts implemented by individuals to stretch their current capabilities and learn new skills/abilities to facilitate personal growth and environmental mastery” (Chen et al., 2022, pp. 12–13).

*Crafting within and across life domains* is described as a motivated process, including goal-directed initiation and engagement in crafting efforts to satisfy psychological needs (De Bloom et al., 2020). It consists of *approach-and avoidance-focused crafting strategies*. *Approach-focused strategies* consist of the “expansion-oriented crafting efforts aimed at approaching or adding desirable aspects of work or nonwork identities” (De Bloom et al., 2020, p. 1424) and include *autonomy* (“the need to decide by oneself which activities to complete”), *competence* (“the need to effectively bring about desired effects and outcomes”), and *relatedness* (“the need to feel close and connected to significant others”; Bindl et al., 2019, p. 606). *Avoidance-focused crafting strategies* are *“*contraction-oriented crafting aimed at avoiding or reducing the negative aspects of work or nonwork roles” (De Bloom et al., 2020, p. 1424). *Avoidance needs* include *detachment* (a subjective experience that goes beyond the pure physical distance from one’s workplace; Sonnentag and Fritz, 2015, p. 74). *Relaxation* (a process often associated with leisure activities is characterized by a state of low activation and increased positive affect; Sonnentag and Fritz, 2007, p. 206), whereas *stress reduction* is the need for strategies that manage one’s reaction to stress and induce feelings of calmness and relaxation.

*Needs crafting* is “the proactive self-management of need-based experiences and entails both awareness of one’s personal sources of psychological need satisfaction and a tendency to act upon this awareness” (Laporte et al., 2021, p. 68). The three dimensions of *need crafting* are *autonomy need crafting, competence need crafting,* and *relatedness need crafting*. *Autonomy need crafting* are activities that allow for a better realization of one’s personal interests, values, and preferences. *Competence need crafting* consists of activities conducive to one’s skill development and emerging mastery. Lastly, *relatedness need crafting* is about creating genuine, reciprocal care and intimate relationships.

Finally, *leader role crafting* “is a conscious, purpose-driven activity aimed at influencing the development of leader roles and exploring how it is interlinked with role identities” (Gjerde and Ladegård, 2019, p. 45). *Leader role crafting* includes leader role identity, personal role definition, and subordinates’ role expectations. *Role-crafting strategies* consist of the steps (1) *present,* (2) *adapt,* (3) *challenge,* and (4) *explore. Present* consists of *inform* (inform subordinates about how they will enact the leader role) and *demonstrate* (show [behaviorally and symbolically] how they interpret the leader role) elements. *Adapt* includes *comply* (compliance to subordinate’s leader role expectations) and *moderate behavior* (alter behavior to meet subordinates’ leader role expectations) facets. *Challenge* is divided into *persuade* (sell in an attempt to convince subordinates about their own leader role conception) and *oppose* (oppose role expectations to fight for their own leader role conception) components. *Explore* consists of *experiment with old ways* (exploring old ways of enacting the leader role by drawing upon experience from previous roles) and *experiment with new ways* (copying ways of enacting the leader role from role models and improvising with new forms of enacting the leader role).

### Categorization and classification of crafting approaches and behaviors

The extracted data were subjected to conventional content analysis to determine the overlap among various crafting approaches and their underlying behaviors. This involved an iterative process of classification and categorization, which is summarized in Table 2. First, the conceptual overlap between crafting behaviors in different domain-specific crafting strategies was identified and categorized. The results showed that 223 categories of crafting could be extracted from the data. From these crafting categories, 48 elements of crafting could be identified. These elements represented general crafting behaviors that could be subjected to further categorization. Second, the elements were categorized into seven broader themes representing general ‘life-crafting strategies’: cognitive crafting, environmental crafting, relational crafting, resources-demands crafting, skill crafting, and task crafting. Third, for inclusion into our final model of life-crafting, crafting behaviors (or elements) should have been present in at least two of the three broader domain-specific contexts. To simplify the classification process, the crafting behaviors or elements were categorized into three broad domains: crafting at work (job crafting, career crafting, reemployment crafting), crafting at home (home crafting, work-life balance crafting, leisure crafting, off-job crafting), and miscellaneous crafting. For an element to be included in the Holistic Life-crafting Model, it must be prevalent in at least two of the three crafting domains. Table 2 presents the frequency of each crafting behavior in each life domain.

**Table 2:** Content Analysis: Life Crafting Strategies, Behaviors, and Domains

**Theme**	**Element**	**Description**	**Job**	**Home**	**Miscellaneous**	**Total**
Cognitive Crafting	Altering perceptions	Redefine view of tasks	3	-	-	3
	Acceptance	Accepting the conditions of one’s work roles	-	1	-	1
	Identify Formation	Creating a positive self-concept in non-work domains through investing in important non-work identities such as that of a family member, friend or volunteer	3	-	8	11
	Reflective Practices	Reflect on and being mindful about aspirations and motivations	4	-	4	8
	Meta-Cognition	Thinking about the process through which individuals interpret and enact roles and make decisions	3	-	-	3
	Cognitive Detachment	Mentally disengaging from work-related matters	-	1	1	2
	Strategic Risk Taking (Autonomy of Choice)	Viewing (im)balance as a consequence of choice/responsibility	1	-	5	6
	Expanding Perceptions	Widening one's understanding of work's influence/impact or purpose	6	-	-	6
	Cognitive Withdrawal	Offloading responsibility for incidents or critical situations onto colleagues	3	-	-	3
	Focusing Perceptions	Emphasize the positive qualities of work	2	-	1	3
	Linking Perceptions	Mentally connecting certain activities or relationships to interests, outcomes, or elements of identities that are meaningful	3	-	-	3
Environmental Crafting	Crafting Towards Development	The initiatives that employees take to realize their potential by creating developmental opportunities for themselves, such as opportunities to apply their unused knowledge and skills	3	-	-	3
	Boundary Management	Workers manage, compartmentalize, and control time to maintain work-life balance.	9	15	2	26
	Opportunities to Craft	Being conscious of, or seeking opportunities to engage in crafting behavior	1	-	-	1
Interests Crafting	Hobby Participation	Directly engaging in activities outside the work domain to increase one’s sense of joy and meaning	-	2	1	3
	Vicarious Experiencing	Seeking fulfilment by following the involvement of other people	-	2	-	2
	Redesign Interests	Organize work/life so it matches personal interests	5	-	-	5
	Interests Alignment	Dividing tasks/activities between peers to match interests	4	-	-	4
	Adding Interests	Take on more tasks/activities which one enjoys	3	-	-	3
Relational Crafting	Relatedness Crafting	More effectively ensuring the development of relationships characterized genuine, reciprocal care and intimacy	-	-	2	2
	Avoiding Social Demands	Reducing unwanted or draining interactions with individuals at home and work	1	-	-	1
	Building Relationships	Approaches people employ to foster positive, mutually beneficial relationships characterized by feelings of pride, love, dignity, appreciation, and self-worth	5	-	1	6
	Re-frame Relationships	Changing the nature of current relationships to serve a new or more meaningful purpose	5	-	-	5
	Expanding Relationships	Expanding relationships during free time (e.g., meeting colleagues during free time)	10	1	1	12
	Adapting Relationships	Changing the nature and/or function of relationships	2	-	-	2
	Influencing and Negotiating	Persuading others to take over tasks	1	-	-	1
	Managing Social Interactions	Using relationships with friends and family to support and maintain work−life balance	2	5	-	7
Resources-Demands Crafting	Autonomy Crafting	Allow for a better realization of their personal interests	1	1	3	4
	Reducing Hindering Demands	Make work emotionally less intense by reducing those aspects that exceed one’s capabilities	31	8	4	43
	Vitality Management	Taking care of one’s physical and mental health	-	3	2	5
	Optimizing Demands	The simplification or optimization of work processes to make people more efficient (e.g., Traveling business class to work whilst commuting)	-	2	-	2
	Seeking Structural Resources	Actions to increase growth-promoting resources (e.g., requesting flexible working hours)	11	9	4	24
	Seeking Social Resources	Creating social support networks or gaining supervisory feedback or coaching (e.g., asking others at work for advice or feedback)	14	6	4	24
	Increasing Challenging Demands	Initiatives to increase challenging demands in life and at work	8	1	1	10
Skill Crafting	Personal Growth Initiatives	Viewing failures, or mistakes as learning opportunities	1	-	1	2
	Using Skills in Different Ways	Seeking opportunities to use current skills in novel and creative ways	2	-	-	2
	Professional Development Initiatives	Actively seeking training and development opportunities	4	3	2	9
	Competence Crafting	Behaviors associated with skill development and an emerging sense of mastery	-	-	2	2
	Decision Latitude	Autonomy to make independent decisions in the execution of tasks (e.g., deciding how and when to engage in academic activities)	-	-	1	1
	Skill and Strengths Use	Seeking opportunities to use skills, knowledge and strengths to the fullest in life and at work	11	-	1	12
Task Crafting	Work Organization	Reshaping systems and strategies to organize the tangible elements of work.	2	-	-	2
	Redesigning Tasks	Changing the nature or function of life tasks to make them more meaningful	10	2	-	12
	Prioritize Tasks	Prioritizing specific tasks over others to improve efficiency and task execution	-	-	1	1
	Task Emphasizing	Highlighting important tasks which are already part of a formal job description or life role	2	1	-	3
	Task Expansion	Adding tasks or projects perceived to be meaningful	21	1	-	22
	Task Enlargement	Include elements of work and related activities not originally in the formal job description	3	-	-	3
	Task Avoidance	Avoid risky situations/cases/tasks	1	-	-	1
	Task Delegation	Delegating tasks	1	-	-	1

Numbers represent the frequency of occurrence.

First, *cognitive crafting* refers to how people alter their perceptions of different areas or elements of work and life (Berg et al., 2013; Chen et al., 2022). This crafting theme consists of several elements: *altering perceptions* (redefine the view of life tasks), *acceptance* (crafting work-life balance by accepting the nature of a task or a role), *identity formation* (creating a positive self-concept in non-work domains through investing in important non-work identities), *reflective practices* (reflect on and being mindful about aspirations and motivations), *meta-cognition* (thinking about the process through which employees interpret and enact roles and make decisions), and *cognitive detachment* (disengage mentally from work-related matters). Furthermore, cognitive crafting consists of *strategic risk-taking* (viewing [im]balance as a consequence of choice/responsibility), *expanding perceptions* (cultivating meaning by widening their understanding of their jobs’ influence or purpose), *cognitive withdrawal* (offloading of responsibility for incidents or critical situations), *focusing perceptions* (emphasize the positive qualities), *and linking perceptions* (make use of existing components by mentally connecting certain activities or relationships to interests, outcomes, or elements of identities that are meaningful). Only identity formation, reflective practices, cognitive detachment, strategic risk-taking, and focusing perceptions were present in two or more life domains and were retained within the final model.

Second, *environmental crafting* refers to individuals’ adjustments to their physical work, home, or life environments to cultivate more meaningful life experiences (Dash and Vohra, 2020). The elements of environmental crafting were *crafting toward development* (taking the initiative to realize one’s potential by creating or seeking developmental opportunities), *boundary management* (managing, compartmentalizing, and controlling time to maintain work-life balance), and *opportunities to craft* (being conscious of or seeking opportunities to engage in crafting behavior). Only boundary management was present in two or more life domains and was retained in the final model.

Third, *interests crafting* refers to the behaviors exhibited to expand or engage in activities or hobbies people find interesting (Kooij et al., 2020). This theme includes elements of *hobby participation* (engaging directly in activities to increase a sense of joy), *vicarious experiencing* (seeking fulfillment by following the involvement of other people), *redesigning interests* (organizing work/life to match interests), *interests alignment* (dividing tasks/activities to match interests), and *adding interests* (take on more tasks/activities which one enjoys). Only hobby participation was present in two or more life domains and was retained for the final model.

Fourth, *relational crafting* refers to the behaviors people use to create meaning by changing how, when, and with whom they interact (Berg et al., 2013). Relational crafting includes the elements of *relatedness crafting* (effectively ensuring the development of relationships characterized by care and intimacy), *avoiding social demands* (reducing unwanted or draining interactions with individuals at home and work), *building relationships* (approaches people employ to foster positive, mutually beneficial relationships characterized by feelings of pride, love, dignity, appreciation, and self-worth); *re-frame relationships* (changing the nature of current relationships to serve a new or more meaningful purpose), *expanding relationships* (expanding relationships during free time), *adapting relationships* (changing the nature or function of a relationship), *influencing and negotiating* (persuading others to take over tasks), *and managing social interactions* (using relationships with friends and family to support and maintain work-life balance). Only building relationships, expanding relationships, and managing social interactions were present in two or more life domains and were retained in the final model.

Fifth, *resources-demands crafting* refers to the behaviors associated with optimizing life resources, managing obstructive or hindering life demands, and attempting to avoid unnecessary resource loss (Chen et al., 2022). Resources-demands crafting consists of *autonomy crafting* (allowing for a better realization of personal interests), *reducing hindering demands* (reducing those aspects that exceed one’s capabilities), *vitality management* (taking care of one’s physical and mental health), *optimizing demands* (the simplification or optimization of work processes to make people more efficient), *seeking structural resources* (actions to increase growth-promoting resources), *seeking social resources* (creating social support networks or gaining supervisory feedback or coaching), *increasing challenging demands* (initiatives to increase challenging demands in life and at work). Only optimizing demands were absent in two or more life domains and, therefore, not included in the final model.

Sixth, *skill crafting* is concerned with developing a wide range of skills through seeking out training opportunities, engaging in stretching assignments/projects, minimizing failures by focusing on what one does best, and optimizing performance in one’s area of expertise (Bindl et al., 2019). Skill crafting contains elements of *personal growth initiatives* (viewing failures as learning opportunities), *using skills in different ways* (seeking opportunities to use current skills in novel and creative ways), *professional development initiatives* (seeking out training and development opportunities), *competence crafting* (behaviors associated with skill development and an emerging sense of mastery), *decision latitude* (autonomy to make independent decisions), and *skill and strengths use* (seeking opportunities to use current skills, knowledge, and strengths to the fullest in life and work). Only personal growth opportunities, professional development initiatives, and skill/strengths use were present in two or more life domains and were retained in the final model.

Seventh, *task crafting* describes behaviors people engage in to physically alter the type, number, scope, and nature of tasks they perform at work and in life (Wrzesniewski and Dutton, 2001; Chen et al., 2022). The elements of task crafting are *work organization* (reshaping systems and strategies to organize the tangible elements of life), *redesigning tasks* (changing the nature or function of life tasks to make them more meaningful), *prioritizing tasks* (prioritizing specific tasks over others to improve efficiency and task execution), *task emphasizing* (highlighting important tasks which are already part of a formal job description or life role); *task expansion* (adding tasks or projects perceived to be meaningful), *task enlargement* (including elements of work and related activities not originally in the formal job description), *task avoidance* (avoiding risky situations/cases), and *task delegation* (delegating tasks). Only redesigning tasks, task emphasizing, and task expansion were present in two or more life domains and were retained in the final model.

## Discussion

**Figure 2:**
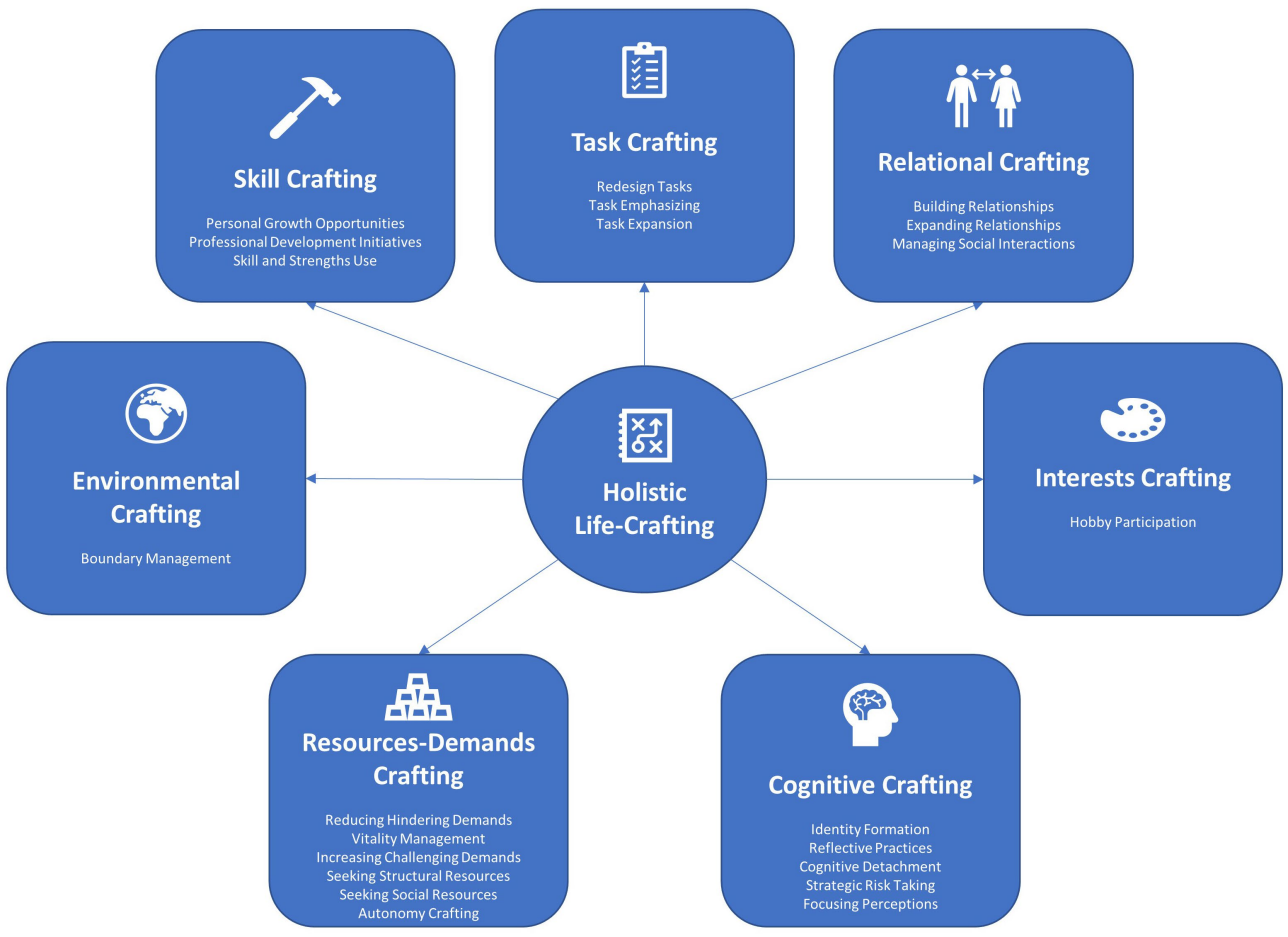
The Holistic Life-Crafting Model.

The present study aimed to advance the theoretical understanding of life-crafting by investigating shared elements or behaviors across different crafting approaches. The review identified 51 records, reflecting five crafting approaches (job crafting, work-life balance and home crafting, leisure crafting and off-job crafting, career crafting, and miscellaneous crafting strategies), comprising 48 different crafting strategies and 223 behaviors. Based on our classification criteria, 22 dimensions were included in the holistic life-crafting model. Content analysis classified these behaviors into seven broader themes representing a general ‘life-crafting’ approach: cognitive crafting, environmental crafting, interest crafting, relational crafting, resources-demands crafting, skill crafting, and task crafting (see Figure 2). The proposed framework comprehensively explains how individuals can actively shape their lives to promote meaningful experiences. The sections below briefly discuss the findings and their implications for future research.

### The Holistic Life-Crafting Model

The first objective was to frame an integrative definition of life-crafting based on the prevailing literature. Results show that life-crafting can be defined as a holistic, continuous process of proactively creating meaning by intentionally balancing demands and resources and altering cognitive, environmental, interest, relational, skill, and task aspects to promote growth and wellbeing. Specifically, this holistic approach indicates that those individuals who actively engage in life-crafting employ seven strategies: cognitive crafting, environmental crafting, interest crafting, relational crafting, resources-demands crafting, skill crafting, and task crafting.

First*, cognitive crafting* refers to how individuals consciously alter the perceptions held or meaning attached to/derived from different areas of work and life. Here, the focus is not on physically changing the nature of life or life-related tasks but rather on the subjective perceptions about work or life (Berg et al., 2013). It encompasses a set of actions taken to create important non-work identities, reflect upon one’s aspirations and motivations, and the practices employed to disengage from work mentally. It further consists of taking strategic risks and emphasizing the positive qualities of work. These activities assist people in seeking, constructing, and experiencing a meaningful existence by increasing their awareness of why their life matters and what they accomplish via their daily actions. When people alter how they think about their lives, it creates a sense of control and mastery, leading to experiences that are perceived to be more meaningful (Berg et al., 2013; Chen et al., 2022). This is in line with the basic tenets of cognitive behavioral therapy, where changes in thoughts, beliefs, and attitudes lead to changes in behaviors, which, in turn, leads to changes in emotional regulation (Thoma et al., 2015).

Second*, environmental crafting* refers to individuals’ physical changes to their work, home, or life environments to cultivate more meaningful life experiences. Specifically, it relates to individuals’ strategies to effectively manage the boundaries between various areas of their lives. Boundary management can lead to more meaningful life experiences by allowing individuals to prioritize their values and goals, establish a sense of control/autonomy over their lives, achieve a better work-life balance, and maintain meaningful relationships with others (Kodama, 2009; Kossek et al., 2012). Given that the environment plays a vital role in both the search for and experience of meaning, it is essential to develop abilities to effectively manage the interaction between different areas of one’s life. Boundary management is a crucial aspect of environmental crafting, which includes managing, compartmentalizing, and governing time to achieve a healthy work-life balance (Wessels et al., 2019). Effective boundary management allows people to prioritize their values and goals and allocate their time and energy accordingly (Kossek et al., 2012). According to Kossek et al. (2012), setting and managing clear boundaries helps people focus on more personally meaningful activities such as spending time with loved ones, engaging in activities that align with their values, or spending time with loved ones. Effective boundary management could also increase the sense of control or autonomy people experience, leading to a greater sense of self-determination and fulfillment (Ryan and Deci, 2000).

Third, *interest crafting* refers to behaviors exhibited to expand or engage in activities/hobbies that people find interesting and meaningful. This includes proactively seeking out, creating, or engaging in enjoyable, fun activities that align with one’s interests and passions (Kooij et al., 2020). When involved in meaningful activities, individuals develop new skills, knowledge, and abilities that contribute to personal and professional development (Van Zyl et al., 2019, 2020). Furthermore, when people engage in interest crafting, there are greater opportunities to enhance self-efficacy. This is because when people engage in activities that are aligned with their interests/passions, they tend to feel more confident in their abilities and more motivated to achieve other goals (Rottinghaus et al., 2003). Further, Kooij et al. (2020) argued that interest crafting could foster creativity as individuals can use their unique skills and perspectives to contribute to their work/lives in new or more innovative ways. This could also lead to a sense of pride or accomplishment as people see the tangible results of their efforts and contributions to their lives (Rottinghaus et al., 2003). Finally, interest crafting can help individuals recharge and re-energize, contributing to overall wellbeing and satisfaction.

Fourth*, relational crafting* refers to the behaviors people use to create meaning by changing how, when, and with whom they interact. It includes behaviors required to create and expand current relationships and those needed to manage personally draining relationships. Research suggests that social relationships play the most important role in creating meaningful life experiences (Steger et al., 2014); thus, building and maintaining social connections are essential for mental health and wellbeing. Relational crafting also encompasses skills and abilities to navigate complex social situations, such as managing conflict and establishing boundaries to maintain better social health and wellbeing (Keyes, 2002). Therefore, by investing in building positive relationships and managing challenging ones, people can create more meaningful connections with others, enhancing their overall sense of purpose and fulfillment.

Fifth, *resources-demands crafting* refers to the behaviors associated with optimizing life resources, managing obstructive or hindering life demands, and attempting to avoid unnecessary resource loss. The underlying elements of resources-demands crafting include reducing hindering demands, vitality management, seeking social resources, seeking challenges, pursuing structural resources, increasing challenging demands, and autonomy crafting. By actively managing one’s resources and demands, people can optimize their use of their resources, manage obstructive demands, and avoid unnecessary resource loss. Research suggests that the availability and use of resources are critical components of the experience of meaning and that resources-demands crafting can help people build and maintain the resources essential for wellbeing (Tims et al., 2012). People experience less stress, conflicts, or pressure when they reduce hindering demands. In this way, people have more energy, which they can invest in other aspects of life that are perceived as meaningful. Proactive vitality management is described as adaptable behaviors that enable people to balance their physical and mental energies (Tisu and Vîrgă, 2022). Improving the balance between physical and mental energies makes people feel more purposeful, leading to a more meaningful life. Increasing structural resources includes opportunities for self-development, autonomy, and resource variety (Yen et al. 2018). These opportunities help people experience a purpose in life and, in turn, more meaning. Increasing social resources is strongly associated with pursuing social support, coaching, or feedback from others (Yen et al., 2018). People may feel more connected, have more in common with others, and have stronger social identities due to developing and maintaining meaningful relationships. Overall, having more access to resources or more opportunities to use resources may facilitate crafting behaviors. This enhances the meaning of life and contributes to an overall sense of purpose in life. Increasing challenging demands refers to the attempts to enlarge the life scope or change the content of tasks (Yen et al., 2018). People develop new skills/abilities and overcome challenges by pursuing difficult goals. Therefore, seeking challenges and increasing challenging demands may result in achievement, progress, and mastery, contributing to their sense of meaning and fulfillment in life. Autonomy crafting is described as pursuing control over one’s actions, life, and choices (Kosenkranius et al., 2020). People may enjoy a higher sense of control, authenticity, and meaning by taking responsibility for their decisions and behaviors.

Sixth, *skill crafting* refers to developing a wide range of skills through seeking out training opportunities, stretching assignments/projects, or minimizing failures by focusing on what one does best and optimizing performance in one’s area of expertise. In essence, skill crafting ensures a closer alignment between the capabilities of the self and the demands/needs/resources of the environment. The underlying elements of skill crafting include personal growth initiatives, professional development initiatives, and skill and strength use. Skill crafting aims to develop new and optimize current skills/abilities to facilitate personal growth and development. Activities that require a more comprehensive range of skills are seen as more meaningful (Berg et al., 2013). People grow more assured, capable, and self-aware when they take the time to develop their skills. These characteristics allow people to take advantage of new chances, work toward worthwhile objectives, and eventually find meaning and fulfillment in their lives. It also aims to develop skills and abilities to facilitate professional development. Persons can expand their knowledge base, develop a higher level of expertise in their industry, and perform better. In response, one’s sense of success and satisfaction increases, boosting confidence and self-esteem. Finally, it aims to facilitate active skills and strengths use to facilitate more meaningful life experiences. Active skill and strength use can improve a person’s sense of competence, self-efficacy, and autonomy, resulting in more fulfilling life experiences. A larger variety of skills and challenges makes tasks and life more meaningful (Li et al., 2020).

Finally*, task crafting* refers to the behaviors people exhibit to physically alter the type, number, scope, and nature of their tasks at work and in life. The underlying elements of task crafting include redesigning life tasks, task emphasizing, and task expansion. Task crafting aims at restructuring tasks or elements of life tasks. It enables people to shape their lives consistent with their values, talents, and interests. It gives people the power to make their lives more personally fulfilling, giving them more meaning and purpose (Hackman and Oldman, 1980). Furthermore, task crafting includes task emphasizing. People craft meaning when engaging in activities that they see as opportunities to gain something valuable (Kosenkranius et al., 2020). Finally, task crafting includes expanding one’s tasks perceived to be more meaningful. Tasks that require a wider range of skills are seen as more meaningful (Berg et al., 2013).

### Similarities and differences to other life-crafting approaches

This holistic approach to life-crafting shares similarities with the Schippers and Ziegler (2019) and Chen et al. (2022) life-crafting approaches in that it aims to present elements required to create more meaningful life experiences and intentionally shape one’s life to align with one’s personal values, interests, needs, and goals. It broadly draws from both the cognitive re-framing and the conservation of resources perspectives on crafting, whereby the focus is on cognitively re-framing life experiences, actively seeking out the means to manage work/life demands, and/or increasing available resources. Furthermore, all three approaches require a deeper level of self-awareness and intentionality as they involve making intentional choices about how to shape experiences and environments. Finally, all three approaches include elements related to increasing and optimizing social aspects of life by highlighting the importance of building and maintaining current and future relationships as routes toward meaning. However, despite these similarities, these approaches have several key differences.

Unlike Schippers and Ziegler (2019) and Chen et al. (2022), our model presents a holistic perspective involving crafting all aspects of life, including nurturing work and home relationships, personal growth, and leisure to create meaning. There are also some global differences among Schippers and Ziegler (2019), Chen et al. (2022), and this approach. All three generally conceive life-crafting as a deliberate process of sculpting and designing one’s life but with a different focus. Schippers and Ziegler (2019) emphasize the psychological components of the process, whereas Chen et al. (2022) emphasize the operationalization and assessment of life-crafting. Chen et al. (2022) developed and established validity evidence for a multidimensional life-crafting measure with three dimensions: cognitive crafting, seeking social support, and seeking challenges. The degree to which people take deliberate action to build a meaningful and fulfilling existence is gaged by this scale. In contrast, Schippers and Ziegler (2019) define life-crafting as a process of looking for meaning and purpose in one’s life and placing more emphasis on its psychological aspects. They contend that the process of ‘life-crafting’ includes ongoing investigation of one’s values, aptitudes, and interests as well as soliciting feedback from others to clarify one’s sense of direction; they stress the significance of taking the initiative to live a life that is consistent with one’s values and aspirations.

The holistic life-crafting model offers clearer insight into the underlying behaviors supporting various life-crafting strategies to synthesize material and offer more comprehensive perspectives on life-crafting. By analyzing the common components and behavioral tactics for diverse crafting strategies, this research seeks to clarify the theoretical underpinnings of life-crafting. Specifically, this paper focused on changing how one thinks (cognitive), functions (environment, resource-demands), acts (task, skill, interest), and fits in (relational) to social contexts. In contrast, Chen et al. (2022) are focused more narrowly on managing resources, and Schippers and Ziegler (2019) on setting goals (aligning values with true self). Despite these global differences, there are also more nuanced differences worth noting.

Whereas Schippers and Ziegler (2019) present life-crafting as an intervention strategy, our life-crafting approach is positioned as an ongoing process whereby an individual continuously reassesses their values, needs, and goals and adjusts their life designs, ensuring better alignment. Furthermore, our approach focuses more on global crafting behaviors and strategies rather than specific solution-orientated tasks such as goal setting, goal attainment, and goal commitment. In contrast to our holistic approach, Schippers and Ziegler (2019) focus on a seven-step process to develop a future or ‘ideal state’ and develop goals required to close the gap between ideal states and the current states. The Schippers and Ziegler (2019) approach seems less about ‘crafting’ *per se* and is more aligned with traditional goal-setting theory, which argues that people are motivated to achieve certain life goals by setting and pursuing mastery and performance objectives (Latham and Yukl, 1975). From this perspective, an ideal state is envisioned, and a clear, actionable plan is created to facilitate the change from the current to the ideal state (Latham and Yukl, 1975). In contrast, holistic life-crafting refers to proactive behaviors required to shape life experiences to align with goals, values, and interests. Furthermore, Schippers and Ziegler’s (2019) approach focuses less on personal agency and meaning and more on improving overall ‘life performance’. Their approach is positioned as a targeted and specific intervention strategy, focusing on achieving personal life outcomes. In contrast, holistic life-crafting is broad, flexible, and focuses on creating more meaningful life experiences.

Similarly, the holistic life-crafting approach also differs from the Chen et al. (2022) framework, focusing on conserving resources (i.e., managing demands/increasing resources) and facilitating growth and development. The Chen et al. (2022) framework heavily relies on the conservation of resources theory. It negates the importance of the environment and the role of tasks, skills, and interests. Furthermore, the Chen et al. (2022) approach arguably provides an oversimplified view of the importance of social relationships in meaning-making, indicating that only relational-seeking behavior is important. In contrast, the findings from this study show that relational crafting has both promotive and preventative components, helping people increase social closeness, build new relationships (and not just seek social support), re-framing the nature of current relationships, and expanding the role of relationships beyond mere functionality. Holistic life-crafting also aims to manage the impact of relationships deemed non-beneficial or ‘draining’ through initiating strategies to avoid social demands and manage social interactions. Only three particular crafting behaviors could be inferred from the questionnaire from Chen et al. (2022): cognitive crafting, seeking social support, and seeking challenges. Our approach included 22 crafting behaviors of life-crafting. Where Chen et al. (2022) used cognitive crafting as a dimension, our holistic model of life-crafting used this type of crafting as a broader theme representing more general “life-crafting” strategies.

### Implications

The holistic life-crafting model proposed in this paper offers several implications for the discipline and practical application. First, the model emphasizes the value of a comprehensive approach to life-crafting by highlighting the overlap of approaches used in different domains and their interconnectedness. Second, the model provides a practical roadmap to help develop a clear strategy for creating meaningful life experiences to guide goal-setting and decision-making. Third, the model highlights the need for a continuous process of self-reflection and assessment to adjust one’s approach over time as circumstances change. Life-crafting can have a positive impact on individuals and organizations, especially on innovation outcomes. By focusing on their personal goals and values, people may become more motivated, engaged, and productive, resulting in more creativity and innovation. Fourth, the model fosters creativity as people use their unique skills and perspectives to contribute to their work/lives in new or more innovative ways. Finally, for organizations, our concept of life-crafting can guide employers and employees in developing a more innovative and empowered culture. People are more likely to contribute fresh viewpoints and original ideas when encouraged to pursue and develop their interests and skills. This may result in a wider variety of solutions and consistent moral and work culture improvements.

### Innovation and future directions

Consolidating the life-crafting literature using an AI-assisted systematic literature review significantly extends the crafting and meaning-making literature. Notably, we used stringent review and evaluation procedures to identify relevant records and evaluate the intersection of these records to promote an inclusive and delineated model (see Figure 2). Our model, Holistic Life-Crafting, pinpoints seven unique dimensions of life-crafting and highlights the unique behavioral expressions of each dimension. In this way, our model successfully consolidated the most relevant literature at the time of evaluation and produced a theoretically grounded framework to help researchers and theorists conceptualize how dynamic forms of crafting fit together. Moving forward, it will be important for researchers to evaluate the structure and formation of our model quantitatively. One unique way of accomplishing this goal is to construct and evaluate a multidimensional assessment tool. Specifically, researchers can design multi-tiered studies to construct and evaluate sets of items to determine the merit of our organizational model. It will be important for researchers to use new-wave psychometric procedures to verify the factor structure and stability of our model across time. To this end, it is recommended that researchers implement modern data driven or exploratory structural equation models (Van Zyl and Ten Klooster, 2022; Van Zyl et al., 2023a) in their studies. Such modeling techniques offer a more accurate representation of how related yet independent dimensions of a construct function together; it assesses how different dimensions intersect in complex models. Assessing intersection elements appears key in life-crating approaches as many dimensions are expected to have dynamic relationships (Chen et al., 2022).

### Limitations and recommendations

Despite efforts to conduct a thorough and comprehensive systematic literature review, a few limitations are worth noting. Although we employed various methods to ensure the inclusion of all potentially relevant texts and followed best practices for systematic reviews, some manuscripts may have been overlooked. Also, excluding gray literature may have resulted in a biased view of the crafting types, as popular psychology press books, dissertations, and theses that present alternative perspectives were not included. Future research might consider a more comprehensive literature analysis, including gray literature and non-academic texts. This may provide new insights which could expand the current model. Another potential limitation relates to the novelty of this study’s machine learning-based screening methods. The model was trained on initial predefined data, but there is currently no means to evaluate the margin of error in the model. This resulted in more time-consuming manual data checking to ensure no important records were missing. Additionally, given the magnitude of the study and the available data on different crafting types, the time frame of our approach was limited (1997–2022). Innovations and publications post-2022 were not included and should be considered in future research. Finally, the criteria for constructing the final holistic life-crafting model may pose challenges. In the current study, we decided to include elements that were present in at least two life domains. However, other elements may also be used in various life domains that science has not yet explored.

## Conclusion

The holistic life-crafting model offers an integrative approach to support individuals in crafting meaningful life experiences by using strategies across various areas of their lives. Our results suggest considerable overlap in the behaviors and strategies people exhibit to craft meaning in different life domains, which signifies the universality of such a concept. Unlike domain-specific approaches, like job crafting or leisure crafting, holistic life-crafting highlights the importance of considering multiple dimensions of an individual’s life to pursuit purpose, meaning, and wellbeing.

## Data availability statement

The raw data supporting the conclusions of this article will be made available by the authors, without undue reservation.

## Author contributions

LZ: Conceptualization, Data curation, Formal analysis, Investigation, Methodology, Project administration, Software, Supervision, Visualization, Writing – original draft, Writing – review, editing. NC: Data curation, Formal analysis, Investigation, Software, Writing – original draft, Writing – review, editing. BD: Conceptualization, Investigation, Methodology, Supervision, Writing – original draft, Writing – review, editing. JK: Conceptualization, Investigation, Methodology, Supervision, Writing – original draft. LV: Writing – review, editing.
