# Supplementary material for: The Holistic Life-Crafting Model: a systematic literature review of meaning-making behaviors
Source: Front Psychol. 2023 Nov 23;14:1271188. doi: 10.3389/fpsyg.2023.1271188 (PMC10701914; doi:10.3389/fpsyg.2023.1271188)
Supplement: Supplementary file 1 [file Table_1.docx]

**Appendix A.** Job Crafting Types, Definitions, and Dimensions.

| **Authors (Year)** | **Crafting type** | **Dimensions of crafting** |
| --- | --- | --- |
| Berg, Dutton, & Wrzesniewski (2013) | **Job crafting**  A self-initiated process of redefining or reimagining job designs in personally meaningful ways. It involves proactively reshaping the boundaries of job-related tasks, relationships and ways of thinking about work | **Task Crafting:** Altering the set of responsibilities prescribed by a formal job description, by adding or dropping tasks, altering the nature of tasks, or changing how much time, energy, and attention are allocated to various tasks. Involves three elements:   1. *Adding tasks*: Adding tasks or projects perceived to be meaningful. 2. *Emphasizing tasks*: Allocating more time to perceptively meaningful tasks. 3. *Redesigning tasks*: Changing the nature or function of tasks to make them more meaningful. |
|  |  | **Relational Crafting:** Changing how, when, or with whom employees interact in executing their jobs. Involves three elements:   1. *Building Relationships*: Establishing new relationships. 2. *Re-framing Relationships*: Changing the nature of current relationships to serve a meaningful purpose. 3. *Adapting Relationships*: Providing help and support to others in executing their duties. |
|  |  | **Cognitive Crafting:** Altering views of the nature and function of tasks, relationships and jobs as a whole. Changing subjective experiences of work, without physically changing elements of work. Involves three elements:   1. *Expanding Perceptions*: Broadening perceptions of the impact or purpose of one’s work. 2. *Focusing Perceptions*: Consciously narrowing the scope of one’s work to specific tasks/relationships deemed significant or valuable. 3. *Linking Perceptions*: Drawing mental connections between specific tasks/relationships and interests, outcomes or aspects of one’s identity that are deemed meaningful. |
| Berg, Grant, & Johnson (2010) | **Job crafting**  Active changes to the behavioral-, relational-, and cognitive boundaries of jobs to alter experiences of- and identities at work. It involves placing more emphasis on essential tasks, adding additional tasks that are deemed meaningful and altering perceptions of one’s work-role to increase enjoyment and meaning at work. | **Task Emphasizing:** Highlighting important tasks which are already part of a formal job description. It involves: (a) changing the nature of job tasks to incorporate aspects of one’s unanswered calling and (b) dedicating additional time, energy, or attention to an assigned task that is related to an unanswered calling.  **Job Expanding:** Adding additional tasks that are deemed to be meaningful through (a) taking on short-term or temporary tasks or (b) increasing the number of tasks continually expected of an individual to incorporate aspects of an unanswered calling.  **Role Re-framing:**  Altering perceptions of the meaning of ones’ work by either (a) establishing a cognitive connection to align the conventional social purpose of a job responsibility with an unanswered calling or (b) broadening the conventional social purpose of a job responsibility to incorporate an unanswered calling. |
| Bindl, Gibson, Unsworth, & Stride (2019) | **Job crafting**  To meet their own individual needs, employees may be required to redesign their jobs under their own initiative by changing the task itself, the way they think about it, the skills they use at their work, or the relational boundaries in their jobs. These changes can be *promotive* (i.e. adding to the scope of work) or *preventative* (i.e. limiting negative outcomes). | **Promotion-Oriented Job Crafting:** Represents a ‘gains approach’ whereby aspects of the job are added or extended to create meaningful work experiences. It involves *Promotion-Oriented*:   1. *Task Crafting***:** Efforts to seek new projects and gains in one’s job by increasing the complexity of tasks or increasing the scope of decisions. 2. *Skill Crafting***:** Gaining a wide range of skills through seeking out training opportunities or engaging in stretching assignments/projects. 3. *Relationship Crafting***:** Advancing meaningful contact and expanding personal networks through building a more comprehensive range of relationships at work. 4. *Cognitive Crafting***:** Changing how jobs are viewed and determining how jobs contribute to the broader organizational context.   **Prevention-Oriented Job Crafting:** Active changes to aspects of a job to limit or prevent negative outcomes from occurring. This does not constitute a withdrawal from work but rather proactive behaviors. It involves *Prevention- Oriented*:   1. *Task Crafting***:** Decreasing experiences of ‘multi-tasking’ and managing productivity losses by putting more effort into aspects of the job that are deemed more important. 2. *Skill Crafting***:** Minimizing failures by focusing on what one does best and optimizing performance in one’s area of expertise 3. *Relationship Crafting***:** Increasing psychological safety by only building relationships with a trusted few or deepening the relationship with valued colleagues.   d. *Cognitive Crafting***:** Focusing on the best parts of a job which make one feel safe and comfortable. |
| Biron, Casper, & Raghuram (2023) | **Role-Based** **Job Crafting**  Shaping work activities and experiences to create conditions for optimal in-role functioning through (a) defining and framing what a job means to one’s identity, (b) managing the quality, scope and location of job-related tasks, and (c) managing social interactions with people at work. | **Cognitive Crafting:** Defining and framing what a job means to one’s identity. It involves shaping or framing:   1. *Work Identity***:** Shaping ones work identity **through e**ngaging in activities to affirm positive self-concepts as contributing members of an organization and finding new ways to stay connected with partners at work. 2. *Non-Work Identity*: Changing the view of self and creating a positive self-concept in non-work domains through investing in important non-work identities such as that of a family member, friend or volunteer. |
|  |  | **Physical Crafting:** Active efforts to maintain work-nonwork boundaries and task allocation through managing the quantity, scope, and location of job tasks. It involves:   1. *Work-Nonwork Boundary Management:* Strategies employed to build, maintain, arrange or cross physical/psychological boundaries between work and none work. 2. *Task Allocation:* Strategies involved in determining the best location to complete work tasks (home/office) and are guided by task requirements. These include active efforts to leverage the benefits of each location (home/office) in task performance. |
|  |  | **Relational Crafting:** Managing social interactions with people in different life domains. This includes crafting:   1. *Professional Networks*: Strategies involved with building and maintaining mutually beneficial relationships with supervisors, coworkers, customers, mentors and the like. 2. *Personal Networks*: Strategies used to meet unmet social needs at work, outside of the work context. |
| Bruning & Campion (2018) | **Job crafting**  Changes to a job that workers make with the intention of improving the job for themselves, and it may include structural (i.e. physical and procedural), social, and cognitive forms. These activities are (a) self-targeted and beneficial to the individual, (b) volitional and represent conscious changes made to elements of work, (c) deviations of pre-crafted work and represents changes in tasks, social activities, behaviors or cognitive processes, (d) leads to permanent or semi-permanent changes to a job, (e) occurs in-role and during work and (f) can only occur when there are clear job descriptions with specified tasks. Both roles and resources can be crafted. | **Approach Crafting:** Activities that are **a**ctive, effortful, motivated and directed towards problem-focused and improvement-based goals which result from the interpretation and acceptance of challenge stressors, attempts to increase resources or a desire to improve the work experience. It involves Approach Role Crafting (changing one’s role in terms of what one does and who one interacts with at work to improve intrinsic benefits, including work-role expansion and social expansion) and Approach Resources Crafting (changing the structural components of one’s job by acquiring new resources, including work organization, adoption, and metacognition.)  *Approach Role Crafting* is comprised of:   1. *Work-Role Expansion*: The self-initiated enlargement of work roles to include elements of work and related activities not originally in the formal job description. 2. *Social expansion*: Occurs within the social domain of work and involves changing the scope, number and nature of social relationships at work.   *Approach Resource Crafting* is comprised of:   1. *Work Organization*: The active design of systems and strategies to organize the tangible elements of work, which can involve managing behavior and/or physical surroundings. 2. *Adoption:* The active and goal-directed use of technology and other sources of knowledge to alter the job and enhance a work process 3. *Metacognition*: The autonomous task-related cognitive activity involving organization, sensemaking, and the manipulation of one’s own psychological states.   **Avoidance Crafting:** Efforts to evade, reduce or eliminate parts of one’s work to reduce hindering and social demands, reduce task/social boundaries at work, and lead to systematic forms for work withdrawal. It involves avoidance role crafting (changing one’s role in terms of what one does and who one interacts with at work to improve intrinsic benefits by work-role reduction) and avoidance resource crafting (re-configuring the resources within one’s job to reduce job demands and their negative implications by withdrawal crafting)  *Avoidance Role Crafting* is comprised of:   1. *Work-Role Reduction*: Consciously, proactively, and systematically reducing the work role, work requirements, effort expenditures, and/or task accountability.   *Avoidance Resource Crafting* is comprised of:   1. *Withdrawal Crafting*: The systematic removal of oneself, either mentally or physically from a person, situation, or event through changes to the job. |
| Hu, Taris, Dollars, & Schaufeli (2020) | **Job crafting**  The activities in which people engage to proactive re-frame or reshape their jobs, making them experience different kinds of meaning of the work and themselves. This may include (a) Approach promoting behaviors, (b) avoidance crafting behaviors, and (c) job characteristics crafting. | **Approach Promoting Behaviors*:*** Active behaviors to create congruence between personal characteristics and the work environment. Its comprised of:   1. *Increasing Structural Job Resources:* The resources variety, opportunity for development, and autonomy whereas the factor. 2. *Increasing Challenging Demands:* The resources social support, supervisory coaching, and feedback.   **Avoidance Crafting Behaviors:** Attempts to conserve existing psychological energy to avoid further energy drain. Its comprised of:   1. *Decreasing Hindering Demands*: Behaviors of employees that would result in additional, challenging demands. 2. *Reducing Task Boundaries:* Employees reduce tasks they dislike either by reducing the effort expended on particular tasks or by omitting tasks and passing them on to colleagues or subordinates. 3. *Reducing Relational Boundaries:* Includes the rejection of clients/customers with whom the job crafters do not fit dissociate from the people with whom they do not get on well. It also includes the rejection of clients/customers with whom the job crafters do not fit.   **Job Characteristics Crafting:** Adaptive responses to changing environmental conditions by balancing job demands and resources to improve person-job fit or a proactive strategy to seek resources and challenging demands to promote meaning and value at work. Its comprised of:   1. *Task Crafting:* Changing number, scope and type of job tasks. 2. *Relational Resources Crafting:* Changing quality and/or amount of interaction with. 3. *Job Demands Crafting:* Initiatives of employees to decrease/increase their level of job demand.   **Role Identity Crafting:** Altering the scope of work to facilitate a secure and stable social identity through extending- and reducing task and social boundaries  **Cognitive crafting***:* Cognitive adjustment at work when people re-frame or redefine their job cognitively, forming it into a meaningful entity  **Behavioral crafting**: Conscious behavioral efforts aimed at changing the nature of tasks and relationships at work. |
| Kooij, Tims, & Kanfer (2015) | **Job crafting: Older Workers**  The self-initiated changes individuals make in the task or relational boundaries of their work aimed  at improving person-job fit. This includes (a) accommodative crafting, (b) developmental crafting, and (c) utilization crafting. | **Accommodative crafting:** Crafting activities directed toward regulating losses, such as hiring an assistant, delegating low priority responsibilities, looking for other ways to achieve goals, and using a professional network for advice and assistance. Its comprised of:   1. *Decreasing job demands:* (i.e., individual’s active attempts to organize work such that it was the least stressful) 2. *Decreasing social job demands:* (i.e., individual’s active attempts to avoid emotionally challenging situations) 3. *Crafting reduced workload:* (i.e., reducing task responsibilities or seeking help from colleagues to reduce the workload) 4. *Increasing social job resources:* (i.e., individual’s job crafting to maximize feedback from the social context)   **Developmental crafting:** Crafting activities that are directed toward learning new skills or growth, such as taking tough assignments, participating in professional organizations, and regularly attending workshops to sharpen knowledge and skills. Its comprised of:   1. *Expanding job to include additional tasks:* (i.e. activities that supplement or expand job boundaries) 2. *Increasing challenging job demands:* (i.e., individual’s crafting to engage in new activities) 3. *increasing quantitative job demands:* (i.e., individual’s active attempts to create more work for him or herself) 4. *Increasing structural and social job resources:* (i.e., individual’s job crafting to maximize feedback from the social context)   **Utilization crafting:** Crafting activities focusing on utilizing existing skills and knowledge, such as focusing on most interesting tasks that optimize existing knowledge and skills, taking on tasks that activate unused skills and resources, focusing on new attainable goals, and taking on tasks through which one can build meaningful relationships and increase the amount of help and mentoring provided to others. |
| Kooij, Nijssen, Bal, & Van Der Kruijssen (2020) | **Job crafting**  Continuously adapting the job to changing personal preferences, motives, and abilities, will lead to positive worker outcomes and will help older workers to age successfully at work. This may include (a) daily interests crafting and (b) daily work pressure crafting. | **Daily interests crafting:** The self-initiated changes that individuals make in their work to make it more enjoyable. This may include (a) making work more challenging, (b) creating more variety in work-related tasks, or (c) changing the conditions of the job to make it more interesting.  **Daily work pressure crafting:** The self-initiated changes that individuals make in their work to lower their work pressure. This may include actions related to reducing the number of tasks performed, choosing not to engage in tasks associated with the execution of work or completely avoiding taking on additional tasks. |
| Demerouti & Peeters (2018) | **Job crafting**  Regulating one’s job demands and resources to create a working situation that better fits one’s preferences. These crafting behaviors could be (a) expansion- or (b) reduction orientated. | **Expansion-oriented:** Seeking resources and challenges.   1. *Increasing the number or complexity of tasks.* 2. *Increasing the interactions with others.*   **Reduction-oriented:** Reducing the number of complexity of the tasks   1. *Minimizing demands:* Behaviors targeted towards minimizing the emotionally, mentally, or physically demanding aspects of one’s work so that job demands do not exceed employees’ capabilities. 2. *Optimizing demands:* The simplification or optimization of work processes to make them more efficient |
| Dominguez, Dolmans, de Grave, Sanabria, & Stassen (2019) | **Job crafting**  The proactivity of workers to optimize and handle their demands (such as workload and physical, emotional, and organizational demands) and resources (such as support, autonomy, and feedback) to achieve a better fit with the work environment. | **Mechanisms of residents’ job-crafting to enhance their work engagement and persistence in training**   1. *Build trust with supervisors to diminish distance toward them.* 2. *Manage proactively to gain responsibility and increase participation.* 3. *Seek help from peers and the social environment to deal with the demands of the residency.* 4. *See errors and frustrations and their handling as learning opportunities.* 5. *Find a suitable work-life balance.* 6. *Actively search for opportunities to be engaged in challenging surgical tasks.* |
| Kroon, van Veldhoven, & Kooij (2013) | **Job crafting**  Make small adjustments to your own job to better match the wishes and abilities of the individual employee. This includes (a) crafting challenging job demands and (b) crafting reduced workload. | **Crafting challenging job demands:**  Taking on varied, challenging and interesting tasks. This form of job crafting touches on the job crafting dimension *‘increasing challenging task requirements’.*  **Crafting reduced workload:** Shedding tasks because of the workload or calling on colleagues. Reducing workload can be seen as a way to ‘*reduce impeding task demands’*. |
| Kooij, van Woerkom, Wilkenloh, Dorenbosch, & Denissen (2017) | **Job crafting**  Employees’ initiative to adapt their job to their personal strengths and interests. This includes (a) crafting towards strengths and (b) crafting towards interest. | **Crafting towards strengths:** The self-initiated changes that individuals make in the task boundaries of their work to make better use of their strengths. This may include (a) *organizing work so it matches strengths, (b) taking advantage of strengths as much as possible in work, (c) looking for possibilities to do tasks in a way that match strengths, (d) dividing tasks with colleagues to match strengths.*  **Crafting towards interest:** The self-initiated changes that individuals make in the task boundaries of their work to make better use of their interests. Actively look for tasks that match one’s interests. This may include (a) *looking for tasks that match interests, (b) organizing work that you can do what you find interesting, (c) taking on tasks you like (d) starting projects with colleagues that share interests,* and *(e) engaging in new relationships at work to make work more interesting.* |
| Kuijpers, Kooij, & Van Woerkom (2020) | **Job crafting**  Adjusting the job to personal strengths, interests and development. This includes (a) crafting towards strengths, (b) crafting towards interests, and (c) crafting towards development. | **Crafting towards strengths (JC-strengths):** The self-initiated changes that individuals make in the task boundaries of their work to make better use of their strengths. Personal strengths are the characteristics of a person that allow them to perform well or at their personal best and are therefore important personal resources. This may include *(a) changing jobs to use current knowledge and capacities to the fullest, (b) taking on tasks you are good at,* and *(c) taking advantage of strengths as much as possible.*  **Crafting towards interests (JC-interests):** Changing the job in such a way that it matches one’s interests. Interests are powerful personal resources, as they can be viewed as motivational factors that trigger employees to invest their time and energy in the topic of their interest. This may include *(a) taking tasks you like, (b) looking for that match own interests,* and *(c) organizing work that you can do what you find interesting.*  **Crafting towards development (JC-development):** The initiatives that employees take to realize their potential by creating developmental opportunities for themselves, such as opportunities to apply their unused knowledge and skills. This may include *(a) looking for opportunities to use different current skills, (b) looking for tasks through which you can develop yourself,* and *(c) looking for tasks that activate unused knowledge and skills.* |
| Lazazzara, Tims, & de Gennaro (2020) | **Job crafting**  Individuals shape their jobs in a manner that better fits their unique characteristics without changing the core of their work. This may include (a) approach crafting, (b) avoidance crafting, and (c) crafting in other domains. | **Approach task/relation/cognitive crafting:** Toward solving problems, improving the work situation, and accepting and interpreting stressors in a positive way.   1. **Approach task crafting:** Increase the number, scope or types of job tasks.  - *Adding extra tasks, altering the scope or nature of tasks, and developing skills and abilities.* - *Caring moves*: Expanded their job by making conversation or “entertaining” patients and directly practising healthcare which was not prescribed in their job. - *Maintaining and upgrading their qualifications.* - *Developing oneself professionally:* Developing skills and knowledge to address work situations more effectively. - *Work role expansion:* Incumbents’ change their tasks by including elements not originally prescribed in the job description and integrating personal and work domains. - *Work organization:* Addresses reshaping systems and strategies to organize the tangible elements of work. - *Prioritizing tasks:* Prioritizing specific tasks over others to improve efficiency and task execution. - *Innovation practices:* Undertake new activities to generate greater involvement. - *Recognition of new business ideas.* - *Adoption:* The use of technology or other information systems to change the work process.  1. **Approach relation crafting:** Increase the quality and/or quantity of interactions with others at work.  - *Actively changing relationships with others at work, creating additional relationships.* - *Social expansion:* Workers’ attempt to systematically seek feedback, change interactions with others or take on self–adopted team roles - *Asking colleagues for feedback and advice, building personal relationships* with partners, and *Creating a network.* - *Seeking emotional and instrumental support* and *persuading others to take over tasks* or re-coordination of projects.  1. **Approach cognitive crafting:** Changing the way one views the job.  - *Redefining their view of the type/nature of tasks or relationships involved in their own jobs.* - *Re-framing their own jobs as a meaningful whole that positively impacts others rather than remaining a collection of separate tasks.* - *Focus on the impact on other people’s lives or on overall organizational success.* - *Changing their mental relationships* and *making one’s work emotionally less intense.* - *Metacognition:* Sensemaking, organization, and the manipulation of one’s own psychological states.   - *Stakeholder prioritization:* Prioritization of certain groups over others when making decisions.   - *Construction of fairness* Concerning beliefs about fairness through which employees interpret and enact their roles and make decisions.   - *Emphasize the positive qualities of work.*   **Avoidance crafting:** Helps to clarify the overall pattern of when which forms of job crafting are utilized by employees and what results these employees experience following this type of job crafting. Avoidance crafting seeks to reduce or eliminate aspects of the job.   1. **Avoidance task crafting**: Reducing the number of tasks, responsibilities, requirements, and effort expenditures.  - *Reducing one’s workload* and *reducing non-critical or non-routine tasks.* - *Avoiding risky situations/cases* and *Delegating tasks.* - *Work role reduction***:** Involves managers delegating tasks or formal responsibilities because they want to avoid a specific task. - *Rule-bound interpretation* of their jobs: Implies strictly applying formal rules and procedures at work and being inflexible or closed to any exceptions.  1. **Avoidance relational crafting**: Reduce the quanity of interactions with others at work.  - *Decrease meetings and time devoted to socializing with colleagues.* - *Social reduction:* Try to reduce unnecessary interactions at work to help them achieve work-life balance. - *Reduce interaction:* Self-protection in risky situations by not disclosing or reporting critical information and reducing interaction with management and coworkers can be a strategy to save time and be more efficient with customers.  1. **Avoidance cognitive crafting:** Individuals use the traditional form of cognitive job crafting by accepting a situation or reducing cognitive demands. Two new forms:  - *Withdrawal crafting:* Involves distancing oneself either mentally or physically from a person, situation, event, or environment. - *Offloading of responsibility* for incidents or critical situations onto colleagues.   **Crafting in other domains:** Refers to “other domains” because it is related to broader aspects of a job, such as the spatial (i.e., where to perform the job) and temporal (i.e., when to perform the job) dimensions or life spheres (e.g., leisure time, work-life balance) that are not strictly work-related.   - *Choosing a job.* - *Locational crafting:* Employees’ managing where to spend work time by either opting to conduct some activities at home to improve work-life balance and wellbeing or preferring to stay at work even when the task can be performed at home to cooperate with colleagues. - *Reducing travel time, prioritizing work,* and *Defining work-life balance crafting.* - *Invest more time and effort in relationships with family and friends* and m*eeting colleagues during free time.* - *Temporal crafting:* The way people manage their workloads during the day. - *Leisure crafting:* The way in which individuals craft their free time. - *Vicarious experiencing:* Considers seeking fulfilment by the involvement of other people*.* - *Hobby participating:* Involves directly engaging in activities outside the work domain to increase one’s sense of joy and meaning. |
| Lichtenthaler & Fischbach (2016) | **Job crafting**  Job crafting comprises behaviors employees perform on their own initiative to redesign their own jobs. It is a function of promotion- and prevention focused strategies. This includes (a) promotion-focused job crafting and (b) prevention-focused job crafting*.* | **Promotion-focused job crafting:** The focus of employees is on satisfying their needs for growth, advancement, and development   1. *Increasing structural job resources:* I.e. developing new capabilities, learning new things at work, using capabilities to the fullest, and the autononomy for deciding how to do things at work. 2. *Increasing social job resources:* I.e. ask a supervisor to act as a coach, asking for performance feedback, looking to the supervisor for inspiration, ask others for feedback on job performance, and ask colleagues for advice. 3. *Increasing challenging job demands:* I.e. volunteering to take on more responsibility at work or engaging in more challenging tasks or projects. |
|  |  | **Prevention-focused job crafting:** The focus of employees is on security and safety   1. *Decreasing hindering job demands:* I.e.make sure work is mentally less intense, manage work to minimize contact with people whose problems affect you emotionally, organize work to minimize contact with people whose expectations are unrealistic, make sure not to have to make many difficult decisions at work, and organize work to make sure that you do not have to concentrate for too long a period at once. |
| Lyons (2008) | **Job crafting**  Represents work and job change that is largely hidden from management and does not include management in decision making. In brief, employees are choosing to engage in shadow job re-design that may or may not run counter to what management desires. This includes (a) personal skill development, (b) task function, (c) advancing relationships, (d) tactics choices, and (e) maintaining relationships. | Category of job crafting:   1. **Personal skill development:** Active investment in the development of professional skills and abilities required to perform better at work. 2. **Task function:** Changes in tasks that are initiated solely by employees for their own purposes. 3. **Advancing relationships:** Activating behaviors to build new and expand upon important relationships at work. 4. **Tactics choices:** Changes in tactics choices. 5. **Maintaining relationships:** Changes implemented to maintain positive relationships with people at work**.** |
| Melo, Dourado, & Andrade (2021) | **Job crafting**  Job crafting is centered on the worker, his/her personal needs and the subjective effects of his/her own actions. Job crafting is about behavior and cognition. It includes (a) approach crafting and (b) avoidance crafting. | **Approach crafting:** Comprises workers’ efforts “directed toward solving problems, improving the work situation, and accepting and interpreting stressors in a positive way.   1. **Cognitive crafting practices:** Interpreting resources and demands. Re-framing demands into resources (and vice-versa):  - *Resources:* Re-framing the job to highlight resources. - *Demands*: Re-framing the job to highlight demands.  1. **Behavioral crafting practices*:*** *Doing more* (means reiterating a specific task or professional contact more often, broadening the scope of these tasks or contacts or even doing tasks and contacts which are not usually expected in a job*) or differently* (associated with reconfiguring the job so that it is completed using a different set of specific tasks or a different set of professional contacts than what is usual for that job).  - *Resources:* Approach resource crafting. - *Demands:* Approach demand crafting.   **Avoidance crafting:** Seeks to reduce or eliminate aspects of the job.   1. **Cognitive crafting practices:**  - *Resources*: re-framing the job to downplay resources. - *Demands*: re-framing the job to downplay demands.  1. **Behavioral crafting practices*:*** *Doing less* (the opposite of doing more, including suppressing altogether tasks and contacts which are usually expected in a job)  - *Resources:* Avoidance resource crafting. - *Demands:* Avoidance demand crafting. |
| Nielsen & Abildgaards (2012) | **Job crafting**  A set of proactive behaviors in which employees may engage to shape their work in order to minimize hindering job demands and maximize resources and challenging demands | **Increasing challenging job demands:** Attempts to engage in new activities.  **Decreasing social job demands:** Active attempts to avoid emotionally challenging situations.  **Increasing social job resources:** Attempts **to** maximize feedback from the social context.  **Increasing quantitative job demands:** Active attempts to create more work for him or herself.  **Decreasing hindering job demands:** Active attempts to organize work such that it was the least stressful. |
| Niessen, Weseler, & Kostova (2016) | **Job crafting**  Changes in the task (cognitive, and behavioral) and social boundaries at work. This may include (a) changing job demands, (b) changing job resources, and (c) adjust perspective on work conditions. | **Task crafting:** Involves employees actively moulding the tasks they have to fulfil at work by taking on more or fewer tasks, altering the scope of the tasks, and changing the means of task accomplishment.  **Relational crafting:** The change of the quality and/or amount of interactions with others at work. Employees decide upon who they will interact with more or less intensively while doing the job.  **Cognitive crafting:** Comprises re-framing how employees perceive their job and altering their cognitive representation of the job. |
| Slemp & Vella Brodrick (2013) | **Job crafting**  The ways in which employees take an active role in initiating changes to the physical, cognitive, or social features of their jobs. It is an informal process that workers use to shape their work practice so that it aligns with their idiosyncratic interests and values. | **Task crafting:** Initiating changes in the number of types of activities one completes on the job (E.g. introducing new tasks that better suit one’s skills or interests)  **Relational crafting:** Exercising discretion about whom one interacts with at work (e.g. making friends with people with similar skills or interests).  **Cognitive crafting:** Alter the way individuals view work to obtain a more positive work identity and derive more meaning and purpose from work. |
| Tims & Bakker (2010) | **Job crafting**  A specific form of proactive behavior in which the employees initiates changes in the level of job demands and job resources. Employees play an active role in determining their job demands and job resources. | **Increasing job demands:** May create more challenges at their work when they feel that their job is not offering them enough opportunities to use all their skills. Employees may increase their level of job demands by adding tasks to their jobs, volunteering for interesting project groups, or taking over tasks from their supervisor.  **Decreasing job demands:** Decreasing the level of job demands when these exceed capabilities. **E**mployees may ask colleagues to help them with their tasks or reduce the number of interactions they have with demanding customers or colleagues. In this way, the employee may be able to attain the work goals without too much effort and by staying healthy.  **Increasing job resources:** Increasing those physical, psychological, social or organizational aspects of the job that may be functional in achieving work goals, (2) may reduce job demands and the associated physiological and psychological costs and (3) may stimulate personal growth and development. This includes (a) s*eeking social support* (employees craft more social support from coworkers) and *(b) enhancing job autonomy* (The extent to which a job allows one the freedom to schedule work, make decisions and select the methods used to perform tasks). |
| Tims, Bakker, & Derks (2012) | **Job crafting**  The self-initiated changes that employees make in their own job demands and job resources to attain and/or optimize their personal (work ) goals, | **Increasing structural job resources:** Active efforts to increase job variety, opportunities for development, and autonomy.  **Decreasing hindering demands:** Efforts to reduce aspects or areas at work which drain energy  **Increasing social job resources:** Active efforts to increase social support, supervisory coaching, and performance feedback.  **Increasing challenging demands:** Initiatives of employees to increase their level of job demand. |
| Weseler & Niessen (2016) | **Job crafting**  Employees who craft their jobs change their cognitions regarding their job by pulling it together into a purposeful entity (i.e. cognitive crafting), and redesign their job by changing the amount and quality of executed tasks (i.e. task crafting) and of cultivated relationships (i.e. relational crafting) | **Task crafting:** Re-framing their job from a multiplicity of disconnected tasks into a connected and meaningful entity.   1. Two types:   *1. Extending task boundaries:* Focus or take on additional preferred tasks, such as the organization of special events.  *2. Reducing task boundaries***:** Employees reduce tasks they dislike either by reducing the effort expended on particular tasks or by omitting tasks and passing them on to colleagues or subordinates.  **Relational crafting:** Redesign their job by changing the amount and quality of cultivated relationships.   1. Two types:   *1. Extending relational boundaries:* Adding and intensifying existing relationships with people with whom an employee gets on well.  *2. Reducing relational boundaries***:** Includes the rejection of clients/customers with whom the job crafters do not fit dissociate from the people with whom they do not get on well. It also includes the rejection of clients/customers with whom the job crafters do not fit. |
|  |  | **Cognitive crafting:** Individuals re-frame or redefine their job cognitively, forming it into a meaningful entity. |
| Wrzesniewski & Dutton (2001) | **Job crafting**  The physical and cognitive changes individuals make in the task and relational boundaries of their work. | **Task crafting:** Changing the number, scope and type of job tasks.   1. *Alter the type of job tasks:* Employees create a different job by choosing to do different tasks than prescribed in the formal job. 2. *Alter the number of job tasks:* By choosing to do fewer or more tasks than prescribed in the formal job, employees create a different job.   **Cognitive task crafting:** Changing cognitive task boundaries.   1. *Alter view of work as discrete parts of a whole:* altering how one sees the job (e.g. as a set of discrete parts or as an integrated whole) |
|  |  | **Relational crafting:** Changing quality and/or amount of interaction with.   1. *Alter with whom one interacts at work:* The people with whom one interacts on and off the job play a role in cocreating and sustaining the claims one makes about one’s work identity. 2. *Alter nature of interactions at work:* The way people interacts on and off the job. |
| Zhang & Parker (2019) | **Job crafting Orientation:**  Approach vs avoidance crafting | **Approach crafting:** Effortful and directed actions to seek positive aspects of work. Approach crafting can be either *(a) behavioral* or *(b) cognitive.*   1. **Approach crafting (behavioral):** Seeking and acting to achieve positive aspects. It may include (a) *resource-focused* or *(b) demand-focused.*  - *Approach demands crafting (behavioral):* Actions to increase one’s challenging demands or address hindering demands. - *Approach resources crafting (behavioral):* Actions to gain positive job resources.  1. **Approach crafting (cognitive):** Seeking to achieve positive aspects cognitively. It may include (a) *resource-focused* or *(b) demand-focused.*  - *Approach resources crafting (cognitive):* Re-framing one’s job to gain positive job resources. - *Approach demands crafting (cognitive):* Re-framing one’s demands as either more challenging or less hinderance.   **Avoidance crafting:** Effortful and directed actions to avoid, or escape from, negative aspects of work. Avoidance crafting can be either *(a) behavioral* or *(b) cognitive.*   1. **Avoidance crafting (behavioral):** Escaping and moving away from negative aspects. Can be (a) *resource-focused* or *(b) demand-focused.*  - *Avoidance resources crafting (behavioral****):*** Actions to avoid aspects of the job that lack positive resources. - *Avoidance demands crafting (behavioral****):*** Actions to avoid hindering demands.  1. **Avoidance crafting (cognitive):** Moving away from negative aspects cognitively. Can be (a) *resource-focused* or *(b) demand-focused.*  - *Avoidance resources crafting (cognitive****):*** Re-framing one’s job to avoid or diminish aspects of the job that lack resources. - *Avoidance demands crafting (cognitive):* Re-framing one’s job to avoid the experience of demands. |
| Kooij, De Lange, & van de Voorde (2022) | **Job crafting**  Adjusting the job to personal resources such as employees’ interests, abilities, knowledge, and growth potential over the lifespan. This includes (a) accommodative job crafting, (b) utilization job crafting, and (c) developmental job crafting. | **Accommodative job crafting:** Accommodating or regulating losses in personal resources. It captures crafting activities aimed at regulating a loss in older workers resources and making sure that they do not overuse their resources by reducing physical, cognitive, emotional, and quantitative demands. This may include *(a) making work emotionally less intense, (b) making work mentally less intense, (c) simplifying tasks,* and *(d) changing the way of working.*  **Utilization job crafting:** Aimed at utilizing current personal resources to compensate for losses in other personal resources. By engaging in utilization crafting (a compensation strategy), older workers strive toward maintaining current levels of functioning when faced with challenges due to the ageing process by compensating for losses in their personal resources using other personal resources. This may include *(a) looking for tasks that match interests, (b) ensuring tasks that you enjoy, (c) changing job to make it more interesting, (d) changing job to use current knowledge and capacities to the fullest,* and *(e) taking tasks you are good at.*  **Developmental job crafting:** Aimed at optimizing personal resources by realizing ones growth potential. by engaging in developmental crafting (an optimization strategy), older workers strive toward functioning at higher levels by optimizing and building their personal resources. Hence, developmental crafting refers to crafting activities aimed at realizing older workers potential by creating developmental opportunities for themselves and increasing challenging demands and responsibilities. This may include *(a) looking at tasks through which you can develop yourself, (b) taking tasks from which you can learn, (c) looking for tasks that activate unused knowledge and skills, (d) taking on more responsibilities,* and *(e) looking for opportunities to use different current skills in work.* |
| Petrou, Bakker, & van den Heuvel (2017) | **Job crafting**  Employee behaviors targeted at increasing one’s social job resources, increasing one’s structural resources, and increasing one’s challenging job demands at work | **Increasing social resources:** Gaining access to instrumental and emotional support from others and fulfilling their psychological need for relatedness (e.g., asking others at work for advice or feedback).  **Increasing structural resources:** Creating enriched jobs and a motivating job environment (e.g., increasing one’s learning opportunities or autonomy at work).  **Increasing challenging demands:** By increasing feelings of competence and mastery experiences and by creating a challenging environment that promotes growth and learning (e.g., asking for new tasks and responsibilities). |
| Yen, Tsaur, & Tsai (2018) | **Job Crafting: Tour Leaders**  the proactive changes tour leaders make to balance their job demands and job resources, using their individual abilities and needs to enhance their enthusiasm and performance. | **Increasing structural job resources:** Tour leaders’ proactive utilization of their own job resources. Examples include opportunities for self-development, job autonomy, and resource variety.  **Increasing social job resources:** Tour leaders’ efforts to seek social support, supervisory coaching, or performance feedback from others (e.g., supervisors, colleagues, and tour members).  **Increasing challenging job demands:** Tour leaders’ attempts to expand their own job scope or adjust the content of their escorting tasks in challenging jobs.  **Decreasing hindering job demands:** Tour leaders’ proactive attempts to reduce the content of tour-leading tasks and lower their job’s demands. |
| Rofcanin, Bakker, Berber, Gölgeci, & Las Heras (2019) | **Relational job crafting**  RJC is a form of job crafting (besides cognitive and physical crafting) and refers to modifying the number and extent of interaction one has with others at work. It includes (a) expansion-oriented and (b) contraction-oriented relational job crafting. | **Expansion-oriented relational job crafting:** A way of expanding the type, number and meaning of interactions employees have with coworkers at work. This may include *(a) expanding conversations, (b) carrying out meetings with new colleagues from another division to achieve work target,* and *(c) involving new colleagues in a project.*  **Contraction-oriented relational job crafting:** A way of contracting the type, number and meaning of interactions employees have with coworkers at work. This may include *(a) limiting meetings with a coworker regarding a project* and *(b) reducing the conference calls with colleagues who may not be directly involved in the concerned project.* |
| Roczniewska, Rogala, Puchalska-Kaminska, Cieslak, & Retowski (2020) | **Job crafting self-efficacy**  An individual’s beliefs about their capability to modify the demands and resources of their job to better fit their needs. | **Increasing structural job resources:** Creating opportunities for development at work or expanding their levels of job autonomy.  **Increasing social job resources:** Look for help or advice from their colleagues to better deal with the demands of their job.  **Optimize the levels of job demands:** Optimizing those aspects of the job that require physical or psychological effort, and are therefore associated with physical and psychological costs.   - 1. ***Increasing challenging job demands:*** Although these demands are appraised as stressful, they provide the potential for growth and may positively affect the individual. e.g., introducing new projects in the company.   2. ***Decreasing hindering demands:*** Other job demands serve as a hindrance to effective goal pursuit and therefore, negatively influence an individual. e.g., reducing workload. |
| Van Wingerden & Niks (2017) | **Job crafting**  Employees’ self-initiated actions to optimize their work environment, employees’ actual job crafting behavior may depend on the opportunities they perceive to do so. | **Perceived opportunity to craft (POC):** Employees’ perception of their opportunity to craft their job and may determine whether they will proactively craft their job. This may include opportunities to *(a) vary the type of tasks carried out, (b) adjust the number of tasks carried out, (c) choose who you want to work with, (d) vary your contacts with other people, (e) take on new activities and challenges,* and *(f) change the meaning of your role.* |
| Wessels, Schippers, Stegmann, Bakker, van Baalen, & Proper (2019) | **Time-spatial job crafting**  A context-specific type of job crafting in which employees (a) reflect on specific work tasks and private demands; (b) select workplaces, work locations, and working hours that fit those tasks and private demands; and (c) possibly adapt either their place/location of work and working hours or tasks and private demands to ensure that these still fit to each other thereby optimizing time/spatial-demands fit. | **Reflection:** A deliberate process of thinking about the tasks and private demands and working hours, places, and locations of work available on any particular day (cognitive component).   1. *Tasks and private demands:* Reflection on task and private demands is likely to foster awareness of the requirements of a particular workday and sensitize employees to the nature of each workplace, work location, and working hours. As such, reflection constitutes the cognitive component of time-spatial job crafting. Once employees have reflected, they can more readily engage in selection, which constitutes the behavioral component.   **Selection:** The actual choice of working hours, work locations, and workplaces, which is then likely to play a part in reaching the best time/spatial-demands fit. It increases in importance when employees are working from a workplace inside the central office.  **Adaption:** Performing adaptive behaviors that address changing conditions (timing/location or task choices may be adapted in hindsight). Behaviors such as either changing the workplace, work location or working hours or changing particular tasks/private demands denote illustrations of adapting within the time-spatial job crafting construct. |

**Appendix B.** Work-Life Balance Crafting and Home Crafting Types, Definitions, and Dimensions

| **Authors (Year)** | **Crafting type** | **Dimensions of crafting** |
| --- | --- | --- |
| Dreyer & Busch (2021) | **Work-life balance crafting**  Proactive, goal-oriented and self-initiated activities to shape boundaries and manage WLB in physical, cognitive and relational ways. | **Dyadic WLB crafting:** Characterized by joint decision-making and the mutual support of actions.  Dyadic WLB crafting strategies:  **Physical dyadic WLB crafting:** Describes how work is organized and it entails joint decisions to change and distribute demands.   1. *Optimizing the way of working.* 2. *Creating recovery opportunities***.**   **Cognitive dyadic WLB crafting:** Redefine their concept of WLB and prioritize one life domain.   1. Balancing act between the omnipresence of the business: *Accepting being a business owner.* 2. The need to indulge in recovery phases and valuing of resources: *Emphasizing health, resources and recovery: identified two groups.*   **Relational dyadic WLB crafting:** Managing and using work-related and out-of-work relationships that provide resources, e.g. understanding or support.   1. *Using and providing spousal social support.* 2. *Using and providing (non-spousal) work-related support.* |
| Caringal-Go, Teng-Calleja, Bertulfo, & Manaois (2022) | **Work-life balance crafting**  The proactive utilization by individuals of strategies aimed at managing WLB experiences within and across various life domains. This includes (a) physical, (b) cognitive, and (c) relational work-life balance crafting. | **Physical work-life balance crafting:** Strategies workers use to shape the physical factors that influence WLB**.** Three themes:   1. *Managing time:* How workers managed, compartmentalized and controlled time to maintain WLB. 2. *Managing work tasks:* How workers managed, compartmentalized and controlled work tasks to maintain WLB. 3. *Managing the workspace:* How workers managed, compartmentalized and controlled the workspace to maintain WLB.   **Cognitive work-life balance crafting:** Strategies and practices that workers employ to manage their perceptions of what WLB meant and entailed.   1. *Enforcing health-related self-care practices:* Taking care of one’s physical and mental health. 2. *Embracing the fluidity between work, rest and leisure:* Regard, workers actively engage in reorganizing their perceptions of what work entailed, required, and demanded in order to preserve their own stability and to minimize frictions between their home and work lives.   **Relational work-life balance crafting**: Involves strategies workers employed to manage both work and non-work relationships.   1. *Spending time with family:* Spending time with their loved ones during short breaks and after work hours or adjust their work schedule to accommodate domestic care responsibilities while working. 2. *Virtually connecting with coworkers:* Workers recognize technology’s usefulness in virtual team collaboration, maintaining both horizontal and vertical work relationships. |
| Sturges (2012) | **Work-life balance crafting behavior**  The unofficial techniques and activities that individuals use to share their own work-life balance. | **Physical work-life balance crafting:** Involves crafting the number, scope or type of job tasks.   1. *Temporal crafting:* Managing the length, timing and temporal experience of the working day. 2. *Locational crafting:* Working away from the office to blend work and non-work. 3. *Choosing a job:* Choosing an employer, job or project which facilitates work-life balance. 4. *Reducing travelling time:* Moving to live nearer work to reduce travelling time.   **Relational work-life balance crafting:** Involves managing the quantity and quality of interactions with other people at work in order to reinforce and maintain a desirable work identity   1. *Managing work relationships:* Using relationships with bosses and colleagues to facilitate work-life balance. 2. *Managing out-of-work relationships:* Using relationships with friends and family to support and maintain work-life balance.   **Cognitive work-life balance crafting:** Involves defining and framing perceptions of what a job means and entails.   1. *Defining work-life balance:* Defining work-life balance to make it possible for it to be achieved. 2. *Prioritizing work***:** Justifying spending time at work rather than at home or on other leisure activities. 3. *Making compromises:* Sacrificing an ‘ideal’ work-life balance in return for future benefits. |
| Gravador (2018) | **Work-life balance crafting**  An individual’s subjective appraisal of the accord between his/her work and non-work activities and life. This includes (a) physical, (b) cognitive, and (c) relational crafting. | WLB crafting behaviors identified in the study were categorized according to the typologies of job crafting behaviors: physical, cognitive, and relational crafting**.**  WLB crafting behaviors (individual employee’s strategies) are assumed to capture individual efforts to attain WLB as a goal.  **Physical crafting** are strategies used to manage the temporal experience of work, work location options, employer choice, and travel time requirements.   1. *Working efficiently:* Structuring work effectively by employing time management and process improvement strategies and by working in one’s most productive time. 2. *Using technology***:** Using technology to manage family tasks and nurture relationships with family members. 3. *Protecting private time***:** Managing a clear and distinct temporal boundary between work and non-work activities to avoid work demands during days off or time after working hours. 4. *Limiting work demands:* Reducing work meetings and workload. 5. *Taking time off/ vacation***:** Plotting and maximizing leaves or days off to attend to non-work activities such as leisure and family activities. 6. *Working away from the office***:** Working from home or outside the office with the help of the internet and mobile technology.   **Cognitive crafting**: Techniques used in which individuals personally describe what is WLB for them, justify the work demands, and sacrifice balance for future benefits.  **Relational crafting** makes use of the individual’s relationship with the people at work and at home to juggle the demands of the two domains.   1. *Fostering relationships with others:* Connecting with peers/friends through communication and spending quality time with them by taking a vacation, travelling, attending social functions, or engaging in de-stressing activities together. 2. *Fostering relationships with family:* Spending quality time with family members by dining, communicating, attending school programs, or allotting a rest day with them. |
| Jammaers & Williams (2021) | **Work-life balance crafting**  The satisfaction of and good functioning at work and home, with a minimum of role conflict. | **Physical crafting temporal:** Oriented around controlling the length of a working day.   1. ***Working part-time.*** 2. ***Requesting flexible hours.*** 3. *Reducing workload.* 4. *Non-engagement at work socializing.* 5. *Maintain a strict divide by blocking out time.* |
|  |  | **Physical crafting locational:** strategy, locational crafting, employees change the location of their work or home, in order to cut down the hours needed to get to or physically be present in their standard workplace.   1. ***Requesting teleworking via technology.*** 2. ***Reducing distance from home–work.*** 3. *Upgrading travel class to work whilst commuting.* |
| Demerouti, Hewett, Haun, de Gieter, Rodríguez-Sánchez, & Skakon (2020) | **Home crafting**  The changes that employees make to balance their home demands and home resources with their personal abilities and needs, in order to experience meaning and create or restore their person-environment fit | 1. *Home seeking resources*: Strategies employed at home to increase the availability of the required resources needed to manage home demands and to achieve goals. 2. Home seeking challenges: Seeking new challenging tasks or taking on more responsibilities once home tasks are completed. 3. Home reducing demands: efforts to lessen the emotional, psychological, or physical taxing aspects of home life. |

**Appendix C.** Leisure Crafting and Off-job Crafting Types, Definitions, and Dimensions

| **Authors (Year)** | **Crafting type** | **Dimensions of crafting** |
| --- | --- | --- |
| Kosenkranius, Rink, de Boom, & van de Heuvel (2020) | **Off-job crafting**  Employees’ proactive and self-initiated changes in their non-working lives to satisfy their psychological needs.  It enables restoration of depleted resources during non-working time and employees’ capacity to cope with workplace stressors successfully, thereby preventing negative effects of job demands on employee burnout | **DRAMMA model: 6 psychological needs:**   1. ***Crafting for Detachment*:** Mentally disengaging from work-related matters // “Switching off” from one’s thoughts related to work and tasks during off-job time. 2. ***Crafting for Relaxation*:** Proactively striving for feeling physically well and for reducing effortful activities. 3. ***Crafting for Autonomy*:** Striving for a feeling of being in control over one’s actions, life, and choices. 4. ***Crafting for Mastery*:** Seeking learning opportunities and optimal challenges to experience feelings of achievement and competence. 5. ***Crafting for Meaning:*** Engaging in activities that individuals perceive as opportunities to gain something valuable in life. 6. ***Crafting for Affiliation*:** The desire to experience relatedness and belongingness with other people. |
| Petrou, Bakker, & van den Heuvel (2017) | **Leisure crafting**  Is the proactive pursuit of leisure activities targeted at goal setting, human connection, learning, and personal development.  Is used by employees to compensate for the fact that their work environment may not allow for job crafting. | **Increasing social resources:** Gaining access to instrumental and emotional support from others and fulfilling their psychological need for relatedness (e.g., asking others at work for advice or feedback).  **Increasing structural resources:** Creating enriched jobs and a motivating job environment (e.g., increasing one’s learning opportunities or autonomy at work).  **Increasing challenging demands:** By increasing feelings of competence and mastery experiences and by creating a challenging environment that promotes growth and learning (e.g., asking for new tasks and responsibilities).  When a job provides low opportunities for crafting (e.g., possibilities to reshape one’s task, to seek additional contact with others, or to learn), employees may attach more importance to behaviors in alternative life domains (e.g., leisure crafting) and they may see them in a different light. |
| Tsaur, Yen, Yang, & Yen (2021) | **Leisure crafting**  An actively initiated act whereby individuals balance leisure demands and resources by utilizing their personal abilities and needs. | **Increasing structural leisure resources:** Crafters increase structural resources when they proactively use their leisure-related resources. Leisure resources are resources that are available for achieving leisure goals; reducing leisure opportunity costs; and stimulating personal growth, learning, and development.  **Increasing social leisure resources:** Crafters actively seek leisure-related support, guidance, and performance feedback from others.  **Increasing challenging leisure demands:** Leisure crafters are willing to solve problems caused by unknown factors or personal incompetence; that is, crafters attempt to increase the challenge of leisure activities by joining different activities or by adjusting the content of these activities. Leisure demands such as physical burdens and specific mental traits, tend to continually incur physical and psychological costs. Leisure crafters balance leisure demands with available resources according to their personal interests, knowledge, and skills.  **Decreasing leisure barriers:** Leisure crafters may strive to reduce factors hindering leisure participation (e.g., reducing concerns regarding leisure participation, seeking companions with similar interests, and seeking activities that are achievable for them), thereby strengthening leisure activity participation. |
| Berg, Grant, & Johnson (2010) | **Leisure crafting**  Exercising initiative, agency, and proactivity to create opportunities for experiencing states of enjoyment and meaning associate with pursuing their unanswered callings as formal occupations during leisure time. This involves (a) vicariously living out meaningful experiences through others and (b) engaging in meaningful hobbies. | **Vicarious Experiencing: S**eeking fulfilment through others’ (e.g. family, friends, celebrities) participation in one’s own unanswered calling. This involves meaningful or enjoyable experiences that one usually associates with living out one’s calling in one’s occupation, but through others.  **Hobby Participation**: Pursuing leisure and volunteer activities related to an unanswered calling outside of work |
| Petrou & Bakker (2016) | **Employee leisure crafting**  The proactive pursuit and enactment of leisure activities targeted at (a) goal setting, (b) human connection, and (c)33 learning and personal development. | **Leisure crafting activities**   1. **Goal setting***:* Setting personal goals and creating strategies for actively achieving such through leisure activities. 2. **Human connection:** Increasing social contact with others and implementing strategies to develop new human relations during leisure time. 3. **Learning and personal development:** Seeking growth and development opportunities via leisure activities. |

**Appendix D.** Career Crafting and Reemployment Crafting Types, Definitions, and Dimensions

| **Authors (Year)** | **Crafting type** | **Dimensions of crafting** |
| --- | --- | --- |
| Lee, Chen, Kolokowsky, Hong, Siegel, & Donaldson (2021) | **Career crafting**  A set of proactive and congruence-seeking behaviors that (a) broadens career-relevant resources in response to the evolving nature of jobs and (b) explores career options more congruent to one’s changing needs, values, and interests. | **Career-level task crafting:** The practice of changing the type, scope, and number of job tasks to suit an individual’s strengths and values better.   1. *Expanding task boundaries:* Career crafters are willing to take on extra tasks to experience new career-related responsibilities in their organization.   **Career-level relationship crafting:** To change the amount and quality of interactions with other people encountered on the job.   1. *Changing relational boundaries:* The vital role of proactive relational crafting in producing positive career outcomes 2. *Utilizing relational resources:* The vital role of proactive relational crafting in producing positive career outcomes.   **Career-level cognition crafting:** Crafting involves altering the individual’s perception of their work, such as interpreting their job as a part of fulfilling their life story instead of viewing work as a means of living.   1. *Reflecting positive career meaning:* Indicating that career crafters view their careers as a significant part of their life, suggesting that meaningful careers are important for career crafters. |
| Tims & Akkermans (2020) | **Career crafting**  Proactive behaviors that individuals perform to self-manage their career and that are aimed at attaining optimal person–career fit. | Self-initiated career-related behaviors: Proactive behaviors in career management.  **Proactive career behaviors:** Should allow individuals to achieve life and career success.   1. ***Proactive career reflection:*** Individuals who proactively reflect on their career motivations and skills (e.g., on motivations and qualities). 2. ***Proactive career construction:*** Individuals who proactively try to advance their careers by networking, may be more likely to achieve careers they find fulfilling. (e.g., networking and setting goals). |
| De Vos, Akkermans, & van der Heijden (2019) | **Career crafting**  Individual’s proactive behaviors aimed at optimizing career outcomes through improving person-career fit. Individuals should employ a proactive attitude in which lifelong learning and employability are key pillars and in which both more minor activities in one’s work and career and major transitions form part of the career development process. | Individuals actively craft their careers over time by *(a) Reflecting on and being mindful about their career aspirations and motivation* and *(b) Making choices that can impact both short-term (e.g. work engagement and performance) and long-term success (e.g. objective and subjective career success)* |
| Hulshof, Demerouti, & Le Blanc (2020) | **Reemployment crafting**  The proactive, self-initiated behaviors undertaken by the unemployed to shape the environmental conditions of their job search in a way that enhances the person–environment (P–E) fit during the job search process. This includes (a) seeking resources, (b) reducing hindering demands, and (c) seeking challenging demands. | **Seeking resources:** The individual’s personality, social support, financial resources, and ability to structure one’s time during  unemployment. This includes proactive behaviors such as asking for feedback on a motivation letter or asking for advice on how to apply for a specific job or at a specific organization.  **Reducing hindering demands:** Reducing those aspects of the job search that exceed one’s capabilities. Reducing hindering demands may include behaviors such as trying to make the quest for work emotionally or mentally less intense.  **Seeking challenging demands:** creating more positively interpreted demands to feel motivated to continue one’s job search. It may include proactive behaviors such as learning how to network or how to make an online video résumé. |

**Appendix E.** Miscellaneous Crafting Types, Definitions, and Dimensions

| **Authors (Year)** | **Crafting type** | **Dimensions of crafting** |
| --- | --- | --- |
| Jammaers & Williams (2021) | **Bodily crafting**  The unofficial techniques and activities disabled employees use to work on their bodies and keep fit for both work and non-work purposes in order to better articulate life and work — to better grasp the embodied experience of a neglected group of workers. | **Cognitive crafting:** takes the form of employees redefining what WLB means to them, seeing it as a dynamic rather than a fixed process of shifting priorities with varying interpretations of work and family ‘success’.   1. ***Adapting the definition of success*** 2. *Reprioritizing when facing a work deadline or home milestone.* 3. *Short-term sacrifices for long-term benefits.* 4. *Do not want to have children.* 5. *Postpone maternity.* 6. *Accept lower-status work.* 7. *Pursue leisure activities to counter workplace stress.* 8. *Seeing (im)balance as a consequence of choice/responsibility.*   **Relational crafting:** consists of building good relationships with key people in one’s environment, both inside and outside the workplace, to establish a better balance.   1. *Build a relationship with the line manager.* 2. *Promoting flexibility with own team.* 3. ***Outsourcing domestic work — paid or family assistance.*** 4. *Focused attention on family when at home.* 5. *Socialize with people with similar schedules.* 6. ***Cutting out workplace socializing.*** |
| Körner, Rigotti & Rieder (2021) | **Study crafting**  The proactive changes that students make in their study demands and study resources, and therefore the active influence of the student on his or her study environment. | **Study resources:** Psychological, physical, social, or organizational study-related aspects that contribute to goal achievement, reduce study demands or improve personal development.  **Increasing structural resources:** Behaviors that influence the study’s design such as changing levels of autonomy or variety within the job.   1. *Decision latitude*: Consists of two sub-dimensions:  - *Skill discretion*: Extent to which different skills and creativity are required. - *Decision authority*: Autonomy to make one’s own decisions within the study program.   **Increasing social resources: T**he social aspects of the study, such as searching for social support or feedback.   1. *Social support from lecturers*: To how attentive lecturers are and to what extent they care about their students and help them in their studies. 2. *Social support from fellow students:* Interest, helpfulness, friendliness, and good cooperation with fellow students.   **Limit study demands: T**hose psychological, physical, social, or organizational study aspects that require effort and are associated with mental or physiological costs.   1. *Psychological demands:* Workload and time pressure. 2. *Overload:* Qualification requirements as well as qualitative overload. |
| Schippers & Ziegler (2019) | **Life crafting**  Life-crafting is about (1) finding out what you stand for (i.e., values and passions), (2) finding out how to make it happen (i.e., goal-attainment plans), and (3) telling someone about your plans (i.e., public commitment). | **Discovering values and passion:**  It is important that people pursue a career that is in line with what they find to be “important”, rather than engaging in activities they “like”. Therefore, their passion should be aligned with values that they hold dear. Discovering a harmonious passion instead of an obsessive work passion since harmonious passion was related to positive outcomes and obsessive work passion with negative outcomes.  **Reflecting on current and desired competencies and habits:** In order to achieve a match between values and passion, it is important to become aware of one’s current habits and competencies as a first step in changing/adapting.    **Reflecting on present and future social life:** Research shows that people with a strong social network live longer and are healthier and happier. It seems important that in general, people seek out interaction with others who are supportive and from which they receive energy rather than those that cost energy.  **Reflecting on a possible future career:** In a life-crafting intervention, participants could be asked to think about what they would ideally like to do in their job, and what kinds of people they might be working with, either directly or indirectly. They could be asked to reflect on their education and career and consider what they feel to be important in a job and what their ideal colleagues would be like.  **Writing about the ideal future:** It should be stressed that in the intervention, students formulate goals that they find important, not ones that others (parents, peers, or friends) find important or that are pursued solely for reasons of status.  **Writing down specific goal attainment and “if-then” plans:** It is vital for participants to set down a detailed strategy for how they will achieve their goals. This part of the intervention asks participants about their motivations for their goals and gets them to consider the personal and social impact of those goals. They should also be asked to identify potential obstacles and how to overcome them and monitor progress toward the goals they have set.  **Making public commitments to the goals set:** Participants can either write down a number of goals and make them public or have a photo taken to accompany a public statement. This enhances goal attainment. |
| Chen, van der Meij, van Zyl, & Demerouti (2022) | **Life crafting**  The conscious efforts individuals exert to create meaning in their lives through (a) cognitively (re-)framing how they view life, (b) by seeking social support systems to manage life challenges, and (c) to actively seeking challenges to facilitate personal growth. | **Cognitive crafting:** Individual’s ability to proactively reshape or cognitively re-frame the physical, cognitive or social features of work or life in order for it to be perceived as more meaningful.  **Seeking social support:** The extent to which individuals seek out social support systems and networks to achieve personal/professional goals and aid in managing adversity.  **Seeking challenges:** The active efforts implemented by individuals to stretch their current capabilities and learn new skills/abilities to facilitate personal growth and environmental mastery. |
| De Bloom, Vaziri, Tay, & Kujanpää (2020) | **Crafting within and across Life Domains**  A motivated process including the  goal-directed initiation of and engagement in crafting efforts intended to satisfy psychological needs. Its comprised of avoidance- and approach-focused crafting strategies. | Approach-focused: expansion-oriented crafting efforts aimed at approaching or adding desirable aspects of work or nonwork identities   - *Autonomy:* the need to decide by oneself which activities to complete (Bindl et al., 2019, p. 606) - *Competence:* the need to effectively bring about desired effects and outcomes (Bindl et al., 2019, p. 606) - *Relatedness*: the need to feel close and connected to significant others (Bindl et al., 2019, p. 606)   Avoidance-focused: contraction-oriented crafting aimed at avoiding or reducing the negative aspects of work or nonwork roles   - *Detachment:* a subjective experience and goes beyond the pure physical distance from one’s workplace (Sonnentag & Fritz, 2015, p. 74) - *Relaxation:* a process often associated with leisure activities. It is characterized by a state of low - activation and increased positive affect (Sonnentag & Fritz, 2007, p. 206) - *Stress reduction: the need for a strategies that block the reaction of stress and induce calmness and relaxation.* |
| Laporte, Soenens, Brenning, & Vansteenkiste (2021) | **Need crafting**  The proactive self-management of need-based experiences. Need crafting entails both awareness of one’s personal sources of psychological need satisfaction and a tendency to act upon this awareness. | **Autonomy need crafting:** Allow for a better realization of their personal interests, values, and preferences.  **Competence need crafting:** Conducive to their skill development and emerging sense of mastery.  **Relatedness need crafting:** More effectively ensure the development of relationships characterized by genuine, reciprocal care and intimacy. |
| Gjerde & Ladegård (2019) | **Leader role crafting**  It is a conscious, purpose-driven activity aimed at influencing the development of leader roles and exploring how it is interlinked with role identities. Leader role crafting includes *leader role identity (*seeing/thinking of oneself as a leader, or as having confidence in one’s ability to intentionally engage in leadership), *personal role definition* (own expectations of role), and *subordinates’ role expectations.* | **Role-crafting strategies:**   1. *Present:*  - *Inform:* Inform subordinates about how they will enact the leader role. - *Demonstrate:* Show (behaviorally and symbolically) how they interpret the leader role.  1. *Adapt:*  - *Comply*: Comply to subordinate’s leader role expectations. - *Moderate behavior*: Alter behavior to meet subordinates’ leader role expectations.  1. *Challenge:*  - *Persuade*: Sell in an attempt to convince subordinates about own leader role conception. - *Oppose:* Oppose role expectations to fight for own leader role conception (behaviorally & symbolically).  1. *Explore:*  - *Experiment with old ways*: Explore old ways of enacting the leader role by drawing upon experience from previous roles. - *Experiment with new ways*: Copy ways of enacting the leader role from role models and improvising with new forms of enacting the leader role. |

**Appendix F.** Quality Assessment Check: Descriptive Information for Included Records.

| **Authors** | **Year of Publication** | **Type of Paper** | **Title of Paper** | **Title of Journal or Book** | **Impact factor** | **Citations** | **H-index of CA** |
| --- | --- | --- | --- | --- | --- | --- | --- |
| Berg, Dutton, & Wrzesniewski | 2013 | Theoretical | Job crafting and meaningful work | Purpose and Meaning in the Workplace | NA | 892 | 15 |
| Berg, Grant, & Johnson | 2010 | Qualitative | When callings are calling: Crafting work and leisure in pursuit of unanswered occupational callings | Organizational Science | 4.1 | 989 | 15 |
| Bindl, Gibson, Unsworth, & Stride | 2019 | Quantitative | Job crafting revisited: Implications of an extended framework for active changes at work | Journal of Applied Psychology | 9.9 | 204 | 14 |
| Biron, Casper, & Raghuram | 2023 | Theoretical | Crafting telework: A process model of need satisfaction to foster telework outcomes | Personnel Review | 3.9 | 20 | 28 |
| Bruning & Campion | 2018 | Mixed- Method | A role-resource approach-avoidance model of job crafting: A multi-method integration and extension of job crafting theory | Business Horizons | 7.4 | 430 | 8 |
| Caringal-Go, Teng-Calleja, Bertulfo, & Manaois | 2022 | Qualitative | Work-life balance crafting during COVID-19: Exploring strategies of telecommuting employees in the Philippines | Community, Work & Family | 2.3 | 44 | 6 |
| Chen, van der Meij, van Zyl & Demerouti | 2022 | Mixed- Method | The life crafting scale: Development and validation of a multi-dimensional meaning-making measure | Frontiers in Psychology | 3.8 | 6 | - |
| De Bloom, Vaziri, Tay, & Kujanpää | 2020 | Theoretical | An identity-based integrative needs model of crafting | Journal of Applied Psychology | 9.9 | 89 | 36 |
| De Vos, Akkermans, & van der Heijden | 2019 | Theoretical | From occupational choice to a career crafting | The Routledge Companion to Career Studies | NA | 52 | 39 |
| Demerouti & Peeters | 2018 | Quantitative | Transmission of reduction-oriented crafting among colleagues: A diary study on the moderating role of working conditions | Journal of Occupational and Organizational Psychology | 6.2 | 135 | 116 |
| Demerouti, Hewett, et al. | 2020 | Quantitative | From job crafting to home crafting: A daily diary study among six European countries | Human Relations | 5.7 | 71 | 116 |
| Dominguez, Dolmans, de Grave, Sanabria, & Stassen | 2019 | Qualitative | Job crafting to persist in surgical training: A qualitative study from the resident’s perspective | Journal of Surgical Research | 2.2 | 12 | 23 |
| Dreyer & Busch | 2021 | Qualitative | At the heart of family businesses: How copreneurs craft work-life balance | Journal of Family Business Management | 2.7 | 7 | 3 |
| Gjerde & Ladegård | 2019 | Qualitative | Leader role crafting and the functions of leader role identities | Journal of Leadership & Organizational Studies | 4.8 | 32 | 7 |
| Gravador | 2018 | Mixed- Method | Work-life balance crafting behaviors: An empirical study | Personnel Review | 3.9 | 51 | - |
| Hu, Taris, Dollard, & Schaufeli | 2020 | Theoretical | An exploration of the component validity of job crafting | European Journal of Work and Organizational Psychology | 4.3 | 38 | 13 |
| Hulshof, Demerouti, & Le Blanc | 2020 | Quantitative | Reemployment crafting: Proactively shaping one’s job search | Journal of Applied Psychology | 9.9 | 23 | - |
| Jammaers & Williams | 2021 | Qualitative | Care for the self, overcompensation and bodily crafting: The work life balance of disabled people | Gender, Work & Organization | 5.8 | 25 | 8 |
| Kooij, De Lange, & van de Voorde | 2022 | Quantitative | Stimulating job crafting behaviors of older workers: The influence of opportunity enhancing human resource practices and psychological empowerment | European Journal of Work and Organizational Psychology | 4.3 | 30 | 37 |
| Kooij, Nijssen, Bal, & Van Der Kruijssen | 2020 | Quantitative | Crafting an interesting job: Stimulating an active role of older workers in enhancing their daily work engagement and job performance | Work, Aging and Retirement | 3.7 | 49 | 37 |
| Kooij, Tims, & Kanfer | 2015 | Theoretical | Successful aging at work: The role of job crafting | Aging workers and employee-employer relationship | NA | 178 | 37 |
| Kooij, van Woerkom, et al. | 2017 | Quantitative | Job crafting towards strengths and interests: The effects of a job crafting intervention on person-job fit and the role of age | Journal of Applied Psychology | 9.9 | 307 | 37 |
| Körner, Rigotti, & Rieder | 2021 | Quantitative | Study crafting and self-undermining in higher education students: A weekly diary study on the antecedents | International Journal of Environmental Research and Public Health | - | 11 | - |
| Kosenkranius, Rink, de Boom, & van de Heuvel | 2020 | Quantitative | The design and development of a hybrid off-job crafting intervention to enhance needs satisfaction, wellbeing and performance: A study protocol for a randomized controlled trial | BMC Public Health | 4.5 | 22 | 3 |
| Kroon, van Veldhoven, & Kooij | 2013 | Quantitative | Job crafting en bevlogenheid: Zijn er verschillen tussen teams met een restrictieve dan wel onbegrensde werk context? | Gedrag en Organisatie | 0.5 | 13 | 16 |
| Kuijpers, Kooij, & van Woerkom | 2020 | Quantitative | Align your job with yourself: The relationship between a job crafting intervention and work engagement, and the role of workload | Journal of Occupational Health Psychology | 5.1 | 98 | 4 |
| Laporte, Soenens, Brenning, & Vansteenkiste | 2021 | Mixed- Method | Adolescents as active managers of their own psychological needs: The need crafting in adolescents’ mental health | Journal of Adolescence | 3.8 | 49 | 4 |
| Lazazzara, Tims, & de Gennaro | 2020 | Theoretical | The process of reinventing a job: A meta-synthesis of qualitative job crafting research | Journal of Vocational Behavior | 11.1 | 260 | 7 |
| Lee, Chen, Kolokowsky, Hong, Siegel, & Donaldson | 2021 | Quantitative | Development and validation of the career crafting assessment (CCA) | Journal of Career Assessment | 3.2 | 7 | 7 |
| Lichtenthaler & Fischbach | 2016 | Quantitative | The conceptualization and measurement of job crafting | Zeitschrift für Arbeits- und Organisationspsychologie A&O | 1.2 | 63 | 7 |
| Lyons | 2008 | Qualitative | The crafting of jobs and individual differences | Journal of Business and Psychology | 4.8 | 521 | - |
| Melo, Dourado, & Andrade | 2021 | Conceptual | Reclaiming cognitive crafting: An integrative model of behavioral and cognitive practices in job crafting | International Journal of Organizational Analysis | 2.8 | 14 | - |
| Nielsen & Abildgaards | 2012 | Quantitative | The development and validation of a job crafting measure for use with blue-collar workers | Work & Stress | 6.1 | 302 | 56 |
| Niessen, Weseler, & Kostova | 2016 | Quantitative | When and why do individuals craft their jobs? The role of individual motivation and work characteristics for job crafting | Human Relations | 5.7 | 398 | 33 |
| Petrou & Bakker | 2016 | Quantitative | Crafting one’s leisure time in response to high job strain | Human Relations | 5.7 | 117 | 23 |
| Petrou, Bakker, & van den Heuvel | 2017 | Quantitative | Weekly job crafting and leisure crafting: implications for meaning-making and work engagement | Journal of Occupational and Organizational Psychology | 6.2 | 200 | 23 |
| Roczniewska, Rogala, et al. | 2020 | Qualitative | I believe I can craft! Introducing job crafting self-efficacy scale (JCSES) | PIoS ONE | 3.7 | 9 | 15 |
| Rofcanin, Bakker, Berber, Gölgeci, & Las Heras | 2019 | Quantitative | Relational job crafting: Exploring the role of employee motives with a weekly diary study | Human Relations | 5.7 | 72 | 25 |
| Schippers & Ziegler | 2019 | Theoretical | Life crafting as a way to find purpose and meaning in life | Frontiers in Psychology | 3.8 | 144 | 33 |
| Slemp & Vella Brodrick | 2013 | Quantitative | The job crafting questionnaire: A new scale to measure the extent to which employees engage in job crafting | International Journal of Wellbeing | - | 600 | 19 |
| Sturges | 2012 | Qualitative | Crafting a balance between work and home | Human Relations | 5.7 | 229 | 24 |
| Tims & Akkermans | 2020 | Quantitative | Job and career crafting to fulfill individual career pathways | School to retirement and beyond | NA | 36 | 35 |
| Tims & Bakker | 2010 | Theory | Job crafting: Towards a new model of individual job redesign | SA Journal of Industrial Psychology | - | 1774 | 35 |
| Tims, Bakker, & Derks | 2012 | Quantitative | Development and validation of the job crafting scale | Journal of Vocational Behavior | 11.1 | 2286 | 35 |
| Tsaur, Yen, Yang, & Yen | 2020 | Mixed- Method | Leisure crafting: Scale development and validation | Leisure Sciences | 4.4 | 18 | 48 |
| Van Wingerden & Niks | 2017 | Quantitative | Construction and validation of the perceived opportunity to craft scale | Frontiers in Psychology | 3.8 | 32 | 15 |
| Weseler & Niessen | 2016 | Quantitative | How job crafting relates to task performance | Journal of Managerial Psychology | 3.2 | 130 | - |
| Wessels, Schippers, et al. | 2019 | Theoretical | Fostering flexibility in the new world of work: A model of time-spatial job crafting | Frontiers in Psychology | 3.8 | 107 | - |
| Wrzesniewski & Dutton | 2001 | Theoretical | Crafting a job: Revisioning employees as active crafters of their work authors | Academy of Management Review | 16.4 | 5959 | 36 |
| Yen, Tsaur, & Tsai | 2018 | Qualitative | Tour leaders’ job crafting: Scale development | Tourism Management | 12.7 | 40 | - |
| Zhang & Parker | 2019 | Theoretical | Reorienting job crafting research: A hierarchical structure of job crafting concepts and integrative review | Journal of Organizational Behavior | 6.8 | 520 | 7 |

**Appendix G.** PRISMA 2020 Checklist

| **Section and Topic** | **Item #** | **Checklist item** | **Location where item is reported** |
| --- | --- | --- | --- |
| **TITLE** | | |  |
| Title | 1 | Identify the report as a systematic review. | p. 1 |
| **ABSTRACT** | | |  |
| Abstract | 2 | See the PRISMA 2020 for Abstracts checklist. | p. 1 |
| **INTRODUCTION** | | |  |
| Rationale | 3 | Describe the rationale for the review in the context of existing knowledge. | pp. 4-5 |
| Objectives | 4 | Provide an explicit statement of the objective(s) or question(s) the review addresses. | pp. 6, 10-11 |
| **METHODS** | | |  |
| Eligibility criteria | 5 | Specify the inclusion and exclusion criteria for the review and how studies were grouped for the syntheses. | p. 11-12 |
| Information sources | 6 | Specify all databases, registers, websites, organisations, reference lists and other sources searched or consulted to identify studies. Specify the date when each source was last searched or consulted. | p. 12 |
| Search strategy | 7 | Present the full search strategies for all databases, registers and websites, including any filters and limits used. | p. 12 |
| Selection process | 8 | Specify the methods used to decide whether a study met the inclusion criteria of the review, including how many reviewers screened each record and each report retrieved, whether they worked independently, and if applicable, details of automation tools used in the process. | pp.12-13 |
| Data collection process | 9 | Specify the methods used to collect data from reports, including how many reviewers collected data from each report, whether they worked independently, any processes for obtaining or confirming data from study investigators, and if applicable, details of automation tools used in the process. | pp. 14 |
| Data items | 10a | List and define all outcomes for which data were sought. Specify whether all results that were compatible with each outcome domain in each study were sought (e.g. for all measures, time points, analyses), and if not, the methods used to decide which results to collect. | pp. 13 |
|  | 10b | List and define all other variables for which data were sought (e.g. participant and intervention characteristics, funding sources). Describe any assumptions made about any missing or unclear information. | pp. 13 |
| Study risk of bias assessment | 11 | Specify the methods used to assess risk of bias in the included studies, including details of the tool(s) used, how many reviewers assessed each study and whether they worked independently, and if applicable, details of automation tools used in the process. | p. 14 and Table S1 |
| Effect measures | 12 | Specify for each outcome the effect measure(s) (e.g. risk ratio, mean difference) used in the synthesis or presentation of results. | N/A |
| Synthesis methods | 13a | Describe the processes used to decide which studies were eligible for each synthesis (e.g. tabulating the study intervention characteristics and comparing against the planned groups for each synthesis (item #5)). | p. 14 |
|  | 13b | Describe any methods required to prepare the data for presentation or synthesis, such as handling of missing summary statistics, or data conversions. | p. 14 |
|  | 13c | Describe any methods used to tabulate or visually display results of individual studies and syntheses. | p. 14 |
|  | 13d | Describe any methods used to synthesize results and provide a rationale for the choice(s). If meta-analysis was performed, describe the model(s), method(s) to identify the presence and extent of statistical heterogeneity, and software package(s) used. | p. 14 |
|  | 13e | Describe any methods used to explore possible causes of heterogeneity among study results (e.g. subgroup analysis, meta-regression). | N/A |
|  | 13f | Describe any sensitivity analyses conducted to assess robustness of the synthesized results. | N/A |
| Reporting bias assessment | 14 | Describe any methods used to assess risk of bias due to missing results in a synthesis (arising from reporting biases). | p. 14 |
| Certainty assessment | 15 | Describe any methods used to assess certainty (or confidence) in the body of evidence for an outcome. | NA |
| **RESULTS** | | |  |
| Study selection | 16a | Describe the results of the search and selection process, from the number of records identified in the search to the number of studies included in the review, ideally using a flow diagram. | p. 12-13 |
|  | 16b | Cite studies that might appear to meet the inclusion criteria, but which were excluded, and explain why they were excluded. | N/A |
| Study characteristics | 17 | Cite each included study and present its characteristics. | p. 46-54 |
| Risk of bias in studies | 18 | Present assessments of risk of bias for each included study. | Table S1 (supplementary file) |
| Results of individual studies | 19 | For all outcomes, present, for each study: (a) summary statistics for each group (where appropriate) and (b) an effect estimate and its precision (e.g. confidence/credible interval), ideally using structured tables or plots. | p. 46-54 |
| Results of syntheses | 20a | For each synthesis, briefly summarise the characteristics and risk of bias among contributing studies. | p. 15 |
|  | 20b | Present results of all statistical syntheses conducted. If meta-analysis was done, present for each the summary estimate and its precision (e.g. confidence/credible interval) and measures of statistical heterogeneity. If comparing groups, describe the direction of the effect. | N/A |
|  | 20c | Present results of all investigations of possible causes of heterogeneity among study results. | pp. 15-23 |
|  | 20d | Present results of all sensitivity analyses conducted to assess the robustness of the synthesized results. | N/A |
| Reporting biases | 21 | Present assessments of risk of bias due to missing results (arising from reporting biases) for each synthesis assessed. | N/A |
| Certainty of evidence | 22 | Present assessments of certainty (or confidence) in the body of evidence for each outcome assessed. | N/A |
| **DISCUSSION** | | |  |
| Discussion | 23a | Provide a general interpretation of the results in the context of other evidence. | pp. 29-31 |
|  | 23b | Discuss any limitations of the evidence included in the review. | pp. 32-33 |
|  | 23c | Discuss any limitations of the review processes used. | pp. 32-33 |
|  | 23d | Discuss implications of the results for practice, policy, and future research. | p. 31-32 |
| **OTHER INFORMATION** | | |  |
| Registration and protocol | 24a | Provide registration information for the review, including register name and registration number, or state that the review was not registered. | p. 11 |
|  | 24b | Indicate where the review protocol can be accessed, or state that a protocol was not prepared. | N/A |
|  | 24c | Describe and explain any amendments to information provided at registration or in the protocol. | N/A |
| Support | 25 | Describe sources of financial or non-financial support for the review, and the role of the funders or sponsors in the review. | p. 1 |
| Competing interests | 26 | Declare any competing interests of review authors. | p. 1 |
| Availability of data, code and other materials | 27 | Report which of the following are publicly available and where they can be found: template data collection forms; data extracted from included studies; data used for all analyses; analytic code; any other materials used in the review. | p. 14 |
